# Author Index

**DOI:** 10.1093/schbul/sby048

**Published:** 2018-04-01

Aarts, Henk, S77, S242

Abdel-Baki, Amal, S17

Abdulkadir, Ahmed, S184

Abellanas, Adelina, S237

Abi-Dargham, Anissa, S2, S44, S44, S71

Aboraya, Ahmed, S365

Abramovic, Lucija, S109

Abrams, Daniel, S212

Abt, Markus, S340

Acebillo, Siddarta, S423

Acharya, Bishnu, S405

Adair, Amy, S147

Adams, Rick, S327, S385

Adams, Robert, S284

Addington, Jean M., S231

Addington, Jean, S39, S62, S78, S79, S102, S266, S355, S396, S422

Adery, Laura Hieber, S77, S195, S196, S421

Adorjan, Istvan, S192

Adres, Merav, S306

Agartz, Ingrid, S306

Agarwal, Sri Mahavir, S345, S413

Agbedjro, Deborah, S196

Agerbo, Esben, S85, S87, S167

Agid, Ofer, S267, S354, S415

Ahmed, Anthony, S175

Ahmed, Kinza, S207

Ahmed, Raees, S343

Ahn, Yong Min, S203

Ahsan, Zohra, S104, S413

Ahuir Perez, Maria Isabel, S253

Ahuir, Maribel, S251

Aikawa, Sayaka, S315

Ainsworth, John, S50, S106

Aissa, Amina, S163, S258, S371, S403, S423

Aitchison, Kathy, S295

Ajnakina, Olesya, S58, S59, S84, S98, S166, S246, S373

Ak, Sertac, S345

Akbarian, Schahram, S82

Akded, Berna, S368

Akdede, Berna Binnur, S300, S356

Akgul, Ozge, S300

Al Hadithy, Asmar, S295

Alústiza, Irene, S352

Al-Diwani, Adam, S61

Al-Radaideh, Ali, S180

Alagille, David, S176

Alameda, Luis, S25, S236, S310

Albert, Nikolai, S95, S99, S106, S129, S134

Albertini, Giulia, S20

Albuquerque, Ana, S262

Alemán-Gómez, Yasser, S395

Aleman, André, S89, S177

Aleman, Andre, S238, S260

Alena,Gizdić, S195

Alex, Fornito, S162

Alexaki, Danai, S198

Alexandria, Raissa, S262

Algora, Maria José, S303

Alho, Jussi, S172

Ali, Lilas, S426

Alison Adcock, R., S281

Alison, Lo Wai Fan, S427

Allan, Stuart, S312

Allen, Lauren, S332

Allen, Paul, S28, S174

Allerby, Katarina, S426

Allott, Kelly, S32, S95, S99, S130, S404

Allswede, Dana, S422

Almeida, Valéria, S22, S297

Almodóvar, Carmen, S145, S174

Alolayan, Yazeed, S409

Alonso, Anna, S208

Alonso-Lana, Silvia, S145

Alptekin, Köksal, S300, S356

Alptekin, Koksal, S368

Alsop, Eric, S221

Altamirano, Olivia, S265

Altamura, Carlo, S371

Altamura, Mario, S263, S264

Altavilla, Benedetta, S312, S414

Altinok, Simge, S368

Alvárez, Mar, S212

Alvarez-Astorga, Aldara, S186

Alvarez-Jimenez, Mario, S4

Alves, Tania Maria, S195, S376

Álvarez, Mar, S113

Amaral, David, S126

Amaral, Robert, S182

Amato, Davide, S337

Ameis, Stephanie, S226

Amelsvoort, Therese van, S391

Amina, Aissa, S152

Aminoff, Sofie R., S113

Ammar, Hanen Ben, S163, S258

Amminger, G. Paul, S116, S326

Amminger, Paul, S378

Amodio, Antonella, S263, S264

Amoretti, Silvia, S318

Anık, Yonca, S283

Anand, Ravi, S126, S132

Andersen, Gitte, S424

Anderson, Kelly, S108, S271

Anderson-Schmidt, Heike, S155

Anderssen, Jannicke F., S205

Andrade, Chittaranjan, S405

Andrade, Julio Cesar, S195, S376

Andrea, Alexandra, S264, S353

Andreassen, Anna Krogh, S336

Andreassen, Ole A., S113, S205, S227, S362

Andreassen, Ole, S122

Andreasson, Eva, S305, S407

Andreopoulou, Angeliki, S243, S348

Angelique, Lowrie, S370

Anhøj, Simon, S80, S83, S213

Ania, Fiksinski, S247, S376

Anmella, Gerard, S337

Anna-Karin, Olsson, S263

Anne Emilie, Stürup, S134

Anne-Kathrin, Fett, S40

Annia Rüesch,Ranganadan, S254

Annibale, Luciano, S384

Ansorge, Olaf, S192

Anticevic, Alan, S30, S327

Antonija Slaviček, Sučić, S257

Antonsen, Sussie, S167

Antonucci, Linda A., S185

Antonucci, Linda, S100

Anttonen, Esa, S345

Aparecida, Fachim Helene, S222, S227

Appadurai, Vivek, S85

Appiah-Kusi, Elizabeth, S28

Arad, Michal, S191

Arango, Celso, S49, S50, S226

Arban, Roberto, S380

Arce, David, S424

Arends, Johan, S238

Argolo, Felipe, S112, S164, S286

Argyelan, Miklos, S176, S330

Arias, Barbara, S174, S330, S332

Arias, Víctor, S96

Armando, Marco, S38

Armio, Reetta-Liina, S391

Arnold, Chelsea, S302

Arrojo, Manuel, S398

Arroyo, Miguel Bernardo, S205

Arseneault, Louise, S25, S97, S110

Ash, David, S408

Ashcroft, Darren, S388

Asmal, Laila, S261, S364, S367

Assunção-Leme, Idaiane, S184

Astorga, Aldara Alvarez, S249

Athey, Alison, S374

Atienza, Joy, S339

Atkins, Alexandra, S159

Atkins, Joshua, S296

Atkinson, Samuel, S351

Attademo, Luigi, S363

Attrotto, Maria Teresa, S100

Attwood, David, S274

Aubry, Jean-Michel, S254

Auer, Dorothee, S283

August, Sharon, S190

Austin, Stephen, S163

Avagliano, Camilla, S204, S312, S414

Averbeck, Bruno, S383

Awasthi, Swapnil, S287

Ayşe,Kircali, S419

Ayşegül,Özerdem, S356

Ayesa-Arriola, Rosa, S142, S251, S268, S373, S374, S392

Ayllon, Lourdes, S237

Azechi, Hirotsugu, S419

Azis, Matilda, S81

aan het Rot, Marije, S260

Bär, Karl, S152

Bäuml, Josef G., S283

Baños, Iris, S370

Baalbergen, Heleen, S77, S225

Baccari, Flavia, S426

Bach, Michael, S11

Bachman, Peter, S266

Bachmann, Silke, S115

Badan Ba, Maryse, S388

Badcock, Johanna C., S229

Badcock, Johanna, S365

Badia, Francina, S123

Badre, David, S244

Baek, Ji Hyun, S362

Baeza, Immaculada, S337

Baeza, Inmaculada, S123, S125, S181, S183, S229

Bahk, Won-Myong, S161, S201, S206, S218, S236, S264, S296, S304, S308, S326, S367, S401, S411

Bahk, YongChun, S344

Bak, Maarten, S160

Bak, Nikolaj, S289

Baker, Amanda, S151

Baker, Andrea, S237

Baker, Dewleen, S82

Baker, Elizabeth, S353

Bakija, Ivana, S257

Bakker, Geor, S275, S391

Bakker, P. Roberto, S295, S325

Bakstein, Eduard, S173

Balakrishnan, Narayanaswamy, S189

Balducci, Pierfrancesco, S409

Ball, Hannah, S209

Ballon, Jacob, S17

Balzan, Ryan, S73, S208

Banaszak, Beata, S252

Bang, Minji, S188

Barajas, Ana, S210, S238, S317, S348, S372

Barbeito, Sara, S255

Barber, Anita, S289

Barbera, Daniele La, S75, S258

Barbero, Juan David, S303

Barch, Deanna, S140

Bardell-Williams, Melissa, S162, S270, S376

Bargallo, Nuria, S181

Barker, Gareth J., S81, S89

Barker, Gareth, S51, S174

Barker, Peter, S20

Barnes, Catherine, S30, S250, S387

Barnes, Nicholas, S48

Barnes, Thomas, S131, S148

Barnett, Jennifer, S145, S248, S353

Barone, Annarita, S312, S414

Barret, Olivier, S176

Barrigón, Maria Luisa, S348

Barrigón, Maria Luissa, S238

Barrigon, Maria Luisa, S317, S372

Barron, David, S368

Barron, John, S425

Barron, Rachel, S27

Barrowclough, Christine, S197

Barry, Erica, S92

Bartels, Matthew, S17

Bass, Emily, S360

Bassett, Anne, S247, S376

Basso, Manuela, S193

Basson, Wilna, S200

Batalla, Albert, S183

Batty, Rachel, S138, S260

Baumann, Melissa, S126

Baumann, Philipp S., S15, S236, S279

Baumann, Philipp, S133, S395

Baumann, Philippe S, S25

Baumel, Amit, S267

Baumgartner, Markus, S331

Baune, Bernhard, S74

Bayatmokhtari, Maryam, S2

Beale, Eleanor, S374

Bear, Adam, S147

Bearden, Carrie E., S102, S231

Bearden, Carrie, S39, S43, S78, S79, S266, S396, S422

Beardon, Carrie, S62

Beasley, Clare, S71

Becerra, Juan Antonio, S255

Bech, Per, S341, S342

Beck, Katherine, S68, S330

Becker, Benedikt, S355

Bedi, Gillinder, S43

Bediat, Laurent, S388

Begemann, Marieke, S95, S109

Begemann, Martin, S85

Beilen, Marije Van, S344

Belangero, Sintia I., S266

Belangero, Sintia, S96, S184

Belger, Aysenil, S79, S266

Bell, Imogen, S279

Bell, Morris, S196

Bellomo, Antonello, S263, S264

Belsham, Denise, S313

Ben Ammar, Hanen, S371, S403

Benavides, Caridad, S233

Bendall, Sarah, S4

Benedicto, Crespo-Facorro, S384

Benito, Adolfo, S417

Bennett, Jeffrey, S126

Bennett, Melanie, S264, S353

Benros, Michael Eriksen, S273

Benros, Michael, S85, S116

Benson, Taylor, S47

Bentea, Eduard, S299

Bentsen, Havard, S122

Benzian-Olsson, Natashia, S354

Berardi, Domenico, S123

Berardinelli, Laura, S319

Berberian, Arthur de Almeida, S255

Berberich, Diana, S121

Bercik, Premysl, S190

Berciu, Cristina, S235

Berdún, Jesús, S208

Berdowski, Woutje, S287

Beresniewicz, Justyna, S293

Berg, Akiah O., S113

Berg, David, S305

Berger, Gregor, S99, S116, S132, S228, S378

Berger, Maximus, S116, S228, S326

Berger, Philipp, S195

Berk, Michael, S177, S405, S411

Berman, Karen F., S85

Bernardin, Florent, S169

Bernardini, Francesco, S363, S409

Bernardo, Miguel, S351, S360

Bernardo, Miquel, S222, S318, S332

Bernasconi, Corrado, S371

Bernhard,Müller, S128

Berretta, Sabina, S14, S16, S235

Berry, Alexandra, S156, S388

Berthelot, Nicolas, S230

Bertolino, Alessandro, S83, S85, S100, S116, S193

Bertolucci, Bruno, S161, S266, S375, S375

Besteher, Bianca, S178, S287

Bestmann, Sven, S416

Betül,Abut, S211, S316, S419

Bharath, Rose, S181

Bhatia, Triptish, S63

Bhattacharyya, Sagnik, S27, S28, S105, S154, S259, S384, S413

Bhavsar, Vishal, S67

Bhuknik-Atzil, Ortal, S165

Biagianti, Bruno, S246

Bian, Dayi, S421

Biase, Maria Di, S77, S195, S196 S74

Biedermann, Falko, S135

Biegon, Anat, S65

Bighelli, Irene, S305

Bin, Kwak Yoo, S330

Binder, Elisabeth, S26

Binnewies, Julia, S287

Bioque, Miquel, S222

Birchwood, Max, S98, S156

Bird, Jessica, S335

Birk, Merete, S134

Birkeland, Kåre I., S205

Birnbaum, Michael, S100, S259, S267

Birulés, Irene, S238

Birules, Irene, S372

Bischof, Martin, S254, S331

Bismuth, Corinne, S420

Bitsch, Florian, S195

Bittner, Robert, S28, S30, S250, S387

Bjerregaard, Marie, S387

Blackman, Graham, S72

Blackman, Rachael, S244

Blair, Nicholas, S289

Blanchard, Jack, S264, S353

Blanquer, Ignacio, S424

Blasco, Drew, S54

Blasco, Maria A., S226

Blasi, Giuseppe, S85, S100, S116, S193

Bless, Josef J., S267

Bless, Josef, S113, S261, S306

Blest-Hopley, Grace, S154, S384

Bliksted, Vibeke Fuglsang, S336

Bliksted, Vibeke, S169, S252, S350

Blizovski, Meirav, S324

Bloemen, Oswald, S183, S275, S391

Bloomfield, Michael, S21

Blum, Stefan, S61

Bo Mortensen, Preben, S167

Bo, Qijing, S241, S329

Bobes, Julio, S119, S222, S420

Bobkov, Konstantin, S412

Bocaccio, Hernán, S286

Bodor, Davor, S257

Boehme, Rebecca, S278

Boehnke, Jan, S6

Bogers, Jan, S67, S239, S256

Bohlhalter, Stephan, S184

Bohlken, Marc, S109

Boies, Sébastien, S230

Bojesen, Kirsten Borup, S383

Bojesen, Kirsten, S224, S387

Boks, Marco P.M., S109

Boks, Marco, S82

Bolhuis, Koen, S231, S402

Bolt, Luke, S99

Bonet, Lucia, S200, S424

Bonet, Meritxell Tost, S253

Bonevski, Billie, S151

Bonivento, Carolina, S144, S147, S168

Bonoldi, Ilaria, S56, S81

Booij, Jan, S275, S391

Boot, Erik, S247

Bora, Emre, S300, S356

Bora, Lee, S362

Bordón, Claudia, S150

Borgan, Faith, S291

Borgwardt, Stefan, S102, S144, S147, S199, S209, S328, S331

Borland, Ron, S151

Borlido, Carol, S309

Borsboom, Denny, S269

Bortolato, Marco, S126

Bortolon, Catherine, S359

Bortolotti, Lisa, S159

Bortz, David, S339

Bosco, Francesca M., S319

Bose, Anushree, S345

Bossong, Matthijs, S28

Bottino, Cassio, S378

Boucher, Michael, S18

Bouix, Sylvain, S90

Bourque, Francois, S58

Bousman, Chad, S74, S176, S282, S295

Bowie, Chris, S31, S404

Bowtell, Meghan, S162, S270, S376

Boyd, Adrian, S317

Boyette, Lindylou, S269

Bozaoglu, Kiymet, S137, S246

Brønnick, Kolbjørn, S284, S417

Braš, Marijana, S257

Braff, David, S35

Bralet, Marie-Cécile, S268, S420

Brambilla, Paolo, S144, S147, S209, S328

Brammer, Mick, S413

Bran, Mihai, S160

Brandl, Eva, S218

Brandl, Felix, S283

Brandon, Nicholas, S105

Brandt, Allison, S289

Brandt, Jørgen, S167

Branka Restek,Petrović, S257

Brannigan, Ross, S379

Bray, Signe, S355

Breetvelt, Elemi, S247, S376

Breier, Alan, S61, S63

Brennan, Annie, S192

Brennand, Kristen, S32, S34

Bressan, Rodrigo A., S266

Bressan, Rodrigo, S96, S112, S153, S154, S161, S164, S164, S184, S255, S286, S375, S375

Brian, O’Donoghoe, S162

Brice, Nicola, S339

Brinkman, Loek, S242

Bristow, Greg, S233, S234, S234

Brivio, Paola, S202

Brix, Sophie, S116

Broberg, Brian V., S80

Broberg, Brian, S83, S119, S213, S224

Broder, Michael S., S366

Brodeur, Mathieu, S197

Bromet, Evelyn J., S40

Bronstein, Michael, S147

Brookes, Matthew, S171

Broome, Matthew R., S280

Brophy, Lisa, S151

Broughton, Susan, S233, S234

Broussard, Beth, S409

Brouwer, Rachel, S83

Brown, Eric, S329

Brucato, Gary, S233

Bruggeman, Richard, S117

Brugger, Stefan, S13

Brunoni, Andre, S133

Bryant, Caitlin, S54

Bryce, Shayden, S359, S404

Bryl, Karolina, S315

Bubl, Anna, S11

Bubl, Emanuel, S11

Bucci, Sandra, S197

Buchanan, Robert, S63, S176, S190, S422

Buchsbaum, Monte, S268

Buck, Gabriella, S182

Buclin, Thierry, S236

Budde, Monika, S155

Buil, Alfonso, S85

Buonaguro, Elisabetta Filomena, S204

Buonaguro, Elisabetta, S313

Bureau, Alexandre, S230

Burke, Erin, S235

Burke, Shadie, S272, S426

Burnet, Phil, S254

Burns, Jonathan, S271

Burrer, Achim, S174

Burton, Birgitte Klee, S110, S245, S252, S300

Butcher, Nancy, S247

Butjosa, Anna, S113, S212

Byford, Sarah, S57

Byrne, Enda, S12

Byrne, Kelly, S148

Caan, Matthan, S275, S391

Cabezas, Ángel, S253, S303

Cabungcal, Jan Harry, S1

Cadenhead, Kristen, S396

Cadenhead, Kristin S., S102, S231

Cadenhead, Kristin, S39, S62, S78, S79, S266, S422

Caforio, Grazia, S100

Cagliarini, Daniela, S4

Cagney, Gerard, S53, S72, S74

Cahn, Wiepke, S101

Cai, Helen, S73

Caillies, Stéphanie, S381

Cairns, Murray, S296, S398, S400

Calabrese, Francesca, S202

Calamante, Fernando, S91

Calkins, Monica, S57, S305

Callahan, Jennifer, S40

Calrco, Navona, S176

Calvo, Ana, S89, S255

Calvo, Anna, S183

Camilla, Avagliano, S313

Campbell, Alastair, S1

Campbell, Dónal, S318

Campbell, Donal, S147

Campbell-Meiklejohn, Daniel, S350

Canal-Rivero, Manuel, S374, S392

Cañas, Benjamín, S186

Cañete, Carlos, S157, S424

Cannon, Mary, S53, S58, S72, S89, S109, S147, S181, S318, S379, S379, S402

Cannon, Tyrone D., S78, S231

Cannon, Tyrone, S35, S39, S62, S79, S102, S147, S266, S396, S422

Cano-Domínguez, Pablo, S151

Cao, Hengyi, S79

Capdevielle, Delphine, S359

Cappucciati, Marco, S383

Capuani, Clizia, S412

Capulong, Leslie, S409

Caravaggio, Fernando, S290, S329, S393

Carey, Eleanor, S147, S402

Carletti, Francesco, S50

Carlo, Pasquale Di, S193

Carlos, González-Vivas, S384

Carlström, Eva Lindholm, S223

Carlton, Mark, S339

Carmi, Dana, S54

Carney, Rebekah, S405

Carot, Jose Miguel, S200

Carpenter, William, S190

Carpenter,, William T.,Jr. S5, S37

Carr, Matthew, S388

Carr, Vaughan J., S229

Carr, Vaughan, S347

Carr, Vaughn, S296

Carrilero, Raquel Lopez, S210

Carrillo, Facundo, S43

Carrion, Ricardo, S266

Carroll, Vincent, S176

Carruthers, Sean, S78, S137, S143, S246, S359

Carter, Cameron, S4, S126, S140

Casaus, Laurie, S18

Cascia, Caterina La, S75, S258

Caspi, Avshalom, S25, S97, S110

Castañeda, Carmen, S269, S364

Castelao, Enrique, S254

Castellani, Laura, S104

Castle, David J., S151

Castle, David, S214

Castrén, Eero, S17

Castro, Mariana, S286

Castro-Fornieles, Josefina, S125, S181

Català, Ferran, S237

Catalán, Rosa, S332

Cather, Corinne, S361

Cattaneo, Annamaria, S127

Cavalcante, Daniel Azevedo, S153, S375

Cavalcante, Daniel, S96

Cavallato, Roberto, S196

Cecchi, Guillermo, S43, S76, S100

Cederlöf, Erik, S88

Celiker, Ayce, S345

Cella, Matteo, S196

Ceren,Hıdıroğlu, S356

Cermolacce, Michel, S47

Ceylan, Deniz, S356

Cha, Saetbyeol, S387

Cha, Yun-Ji, S242

Chadha, Radhika, S400

Chah, Chloe, S134

Chaimani, Anna, S94

Chakravarty, Mallar, S182, S390, S393

Chamorro, Jacobo, S237

Chan, Kit Wa, S138, S214, S215, S372

Chan, Nathan, S329

Chan, Raymond C. K., S244

Chan, Raymond, S161, S333, S358, S382

Chan, Sherry, S97

Chan, Suet In, S138

Chan-Golston, Alec, S216, S320

Chana, Gursharan, S74

Chance, Steven Andrew, S192

Chandratilleke, Madhu, S284

Chanen, Andrew, S105

Chang, Eunice, S97, S248, S366

Chang, Trina, S361

Chang, WC, S97

Chang, Wing Chung, S138, S214, S215, S243, S372

Chaparala, Srilakshmi, S298

Charles, Ellis, S120

Charters, Beth, S209

Chat, Saetbyeol, S391

Chatham, Chris, S340

Chaudhry, Imran, S131, S148

Chaumette, Boris, S26

Chavez, Sofia, S290, S329

Chavez-Baldini, UnYoung, S6, S307

Chawner, Samuel, S86

Chee, Michael, S281

Cheer, Joseph, S204

Chekroud, Adam, S157

Chelini, Gabriele, S16, S235

Chen, Chi-Ming, S2

Chen, Eric Y. H., S132

Chen, Eric YH, S243

Chen, Eric, S97, S99, S116, S138, S214, S215, S228, S372, S378

Chen, Jianqing, S176

Chen, Jun, S241

Chen, Kao Chin, S389

Chen, Kehui, S63

Chen, Maxine, S97

Chen, Po See, S389

Chen, Qiang, S85

Chen, Te-An, S188, S189

Chen, Yu-Jen, S186

Chen, Yuxiao, S19

Chesney, Edward, S310

Cheung, Eric F.C., S333, S358

Chevallier, Emmanuel, S268

Chi, Su-Hyuk, S403

Chianca, Camille, S195, S376

Chien, Yi-Ling, S186

Chiliza, Bonga, S364

Chiliza, Bonginkosi, S261, S367

Chiramberro, Marcelo, S345

Chisholm, Katharine, S209, S328, S334, S357

Chiu, Chui-de, S161, S389

Chiu, Sanyin, S138, S214

Choe, Ann, S289

Choi, Joonho, S257, S360

Choi, Kee-Hong, S240, S242, S260, S344

Choi, Sun, S240

Chokran, Cole M., S78

Chon, Eon Sol, S47

Choudhury, Munmun De, S100

Chow, Eva, S247

Chowbay, Balram, S309

Christensen, Anne Marie Raabjerg, S333

Christiani, Camilla J., S245

Christiani, Camilla Jerlang, S110, S252, S300, S336

Christiani, Camilla, S300

Chu, Min-yi, S358

Chu, On Ki, S138, S215

Chung, In Won, S203

Chung, Jun Ku, S290, S329, S393

Chung, Yoonho, S79, S396

Churchill, Madeline J, S20

Ciampoli, Mariasole, S38

Ciantar, Sarah, S368

Ciccarelli, Mariateresa, S414

Cid, Jordi, S238, S317, S348, S372

Cieslak, Kristina, S361

Cihakova, Daniela, S190

Cilia, Jackie, S339

Cinar, Ozan, S84

Ciufolini, Simone, S98

Clapcote, Stephen, S234

Clark, Jeffries, S62

Clarke, Latoya, S370

Clarke, Mary, S89, S109, S379

Cleary, Sean, S259, S409

Clementz, Brett A., S121

Clementz, Brett, S6, S14

Clemmensen, Lars, S103, S334, S336

Cleusix, Martine, S15, S25, S133, S236, S389, S395

Cobo, Jesús, S410

Cobo, Jesus, S370, S423

Cocchi, Luca, S91

Coello, Christopher, S385

Coffman, Brian, S288, S390

Cogo-Moreira, Hugo, S266

Cohen, Neta, S198

Cohen, Reut, S202

Cohen-Woods, Sarah, S347

Cohn, Tony, S413

Colantuoni, Carlo, S85

Colizzi, Marco, S246, S384, S413

Collazzoni, Alberto, S267

Collell, Rebeca García, S423

Collia, Deanna, S339

Collier, Tracey, S81

Collier, Tracy, S383

Collin, Guusje, S104

Collins, Anne, S340

Collip, Dina, S84

Colom, Francesc, S5

Common, Stephanie, S321

Comps, Kristiina, S293

Compton, Michael T., S201

Compton, Michael, S167, S178, S259, S409

Conforti, Marco, S105

Conn, P. Jeffrey, S70, S70

Connell, Melissa, S379

Constantinescu, Cristian, S176

Conus, Philippe, S15, S25, S133, S236, S279, S310, S389, S395

Cookey, Jacob, S153

Cooper, Helen, S37

Cooper, Jonathan, S65

Cooper, Owen, S241

Cope, Zackary, S233

Corbett, Ged, S339

Corcoran, Cheryl, S42, S43, S54, S76, S331

Cordeiro, Quirino, S96, S153, S266, S375

Cordes, Joachim, S343

Cornblatt, Barbara A., S78, S102, S231

Cornblatt, Barbara, S39, S62, S79, S266, S396, S422

Cornea, Emil, S103

Coromina, Marta, S212

Corral, Ricardo, S371

Correll, Christoph U., S266, S341, S342

Correll, Christoph, S86, S93, S108, S206, S207, S236, S333, S341, S363, S375

Corripio, Iluminada, S208, S318

Corsi, Mauro, S412

Corsi-Zueli, Fabiana, S329

Corsi-Zuelli, Fabiana, S227

Cortes-Briones, Jose, S27

Cortizo, Romina, S237

Corvin, Aiden, S141

Cosci, Fiammetta, S76

Costanzi, Monise, S288

Costanzo, Elsa, S286

Costas, Javier, S398

Cotel, Marie, S191

Cotel, Marie-Caroline, S65

Coto, Ana, S119, S420

Cotter, David, S53, S72, S74

Cotter, Jack, S145, S248, S353

Coughlan, Helen, S89, S147

Coughlin, Jennifer, S8

Coull, Jennifer, S47

Courouve, Laurène, S125

Courtet, Philippe, S96

Cousins, Ann, S236

Coutinho, Luccas, S96, S153, S154

Coutrot, Antoine, S249

Covey, Daniel, S204

Covington, Michael, S409

Coyle, Joseph, S1

Crabbe, Frances, S282

Craven, Alex, S302

Craven, Alexander R., S292

Crawford, Trevor, S301

Cremers, Dimitri, S219

Crespo-Facorro, Benedicto , S259

Crespo-Facorro, Benedicto, S92, S142, S184, S251, S268, S276, S373, S374, S392

Cress, Rosemary, S4

Crichton, Jonathan, S408

Crippa, Alessio, S94

Crippa, Jose Alexandre, S28, S297

Crisafio, Anthony, S409

Critchley, Hugo, S173

Crocker, Candice, S295

Croft, Jazz, S377

Cropley, Vanessa, S74, S91, S176

Crosas, Josep Maria, S251

Crossland, Georgia, S323

Crossley, Nicolas, S202, S269, S364

Crump, Francesca, S54

Crush, Eloise, S110

Cryan, John, S59

Cuadra, Meritxell Bach, S395

Cubała, Wiesław, S27

Cucchiaro, Josephine, S114, S239, S353

Cuenod, Michel, S2, S15, S25, S133, S236

Cuesta, Manuel J., S318, S374

Cui, HuiRu, S223, S366

Cui, Xi-long, S333

Cullen, Alexis, S102, S118, S143

Cunningham, Janet, S223

Curtis, Logos, S388

Curtis, Vivienne, S72

Czajkowski, Nikolai, S250

Czamara, Darina, S26

Czepielewski, Leticia, S140, S288

D’Alfonso, Simon, S4

D’Ambrosio, Enrico, S68, S225

D’Ambrosio, Luigi, S204, S312, S414

D’Souza, Deepak, S27, S63

Dadabhoy, Maria, S191

Dahoun, Tarik, S385

Dai, Jing, S331

Dalibor,Karlović, S369

Dalman, Christina, S67, S377

Dalmau, Pere Bonet, S205

Dalrymple, Anthony, S72

Dalton, Caroline, S329

Daly, Eileen, S395

Daly-Ryan, Niamh, S349

Damo-Csorba, Bori, S192

Danese, Andrea, S97, S110

Daniel, David, S131, S135

Dantas, Clarissa De Rosalmei, S349

Dantas, Clarissa, S146, S262, S425

Dantzer, Robert, S48

Dark, Frances, S214, S237

Darrelmann, Ute, S128

Das, Tushar, S79, S82, S90, S102, S170, S179, S179, S199, S373

Daumit, Gail, S12

Dauvermann, Maria M., S325

Dauvermann, Maria, S325

Davey, Christopher G., S105

David, Anthony, S39, S58, S72, S77, S87, S92, S98, S166, S246, S247, S328, S374, S396

Davidson, Michael, S23

Davies, Cathy, S56, S162, S323, S383

Davies, Geoff, S173, S212

Davis, Bethany, S139

Davis, John, S94

Davis, Robert, S236, S320, S341

Dawson, Neil, S233, S234, S234

Dazzan, Paola, S6, S48, S50, S77, S92, S98, S118, S131, S148, S166, S246, S367

De Haan, Lieuwe, S116, S269

De Hert, Marc, S84

De Jong, Steven, S260

De La Fuente-Tomas, Lorena, S119, S420

De la Serna, Elena, S125, S229

DeLisi, Lynn, S34, S194

De Luca, Vincenzo, S290

DeMarshall, Cassandra, S328

De Micheli, Andrea, S162, S383

De Nijs, Jessica, S101

De Pino, Gabriela, S286

DeRosse, Pamela, S176, S422

DeVylder, Jordan, S379

Deakin, Bill, S131, S148

Deakin, John, S48

Dean, Brian, S70

Dean, Derek, S42

Dean, Kimberlie, S229

Decoster, Jeroen, S84

Deenik, Jeroen, S407

Degenhardt, Franziska, S287

Degirmencioglu, Banu, S368

Del Cacho, Núria, S113, S212

Del Re, Elisabetta, S90

Del-Ben, Cristina Marta, S329

Del-Ben, Cristina, S166

Delacretaz, Aurélie, S310

Delespaul, Philippe, S84

Delgado-Alvarado, Manuel, S392

Dellazizzo, Laura, S346

Delucchi, Kevin, S4

Demmin, Docia, S10, S401

Dempster, Kara, S179, S179, S207

Demro, Caroline, S43

Demyanovich, Haley, S190

Deneyer, Lauren, S20

Deng, Ling, S239, S353

Denny, Thomas, S120

Denton, Jennifer, S3

Depasquale, Erica, S299

Derogatis, Leonard, S139

Derom, Catherine, S84

Desai, Priyal, S405

Deserno, Lorenz, S278

Deshpande, Gopikrishna, S170, S181

Deshpande, Smita, S63

Devenyi, Gabriel A., S121

Devenyi, Gabriel, S182

Dey, Avyarthana, S221, S327

de Aguileta, Itziar Riera-López, S113, S212

de Apraiz, Ana, S317

de Bartolomeis, Andrea, S204, S312, S313, S414

de Freitas, Rosana, S371

de Graaf, Ron, S160

de Haan, Lieuwe, S86, S107, S132, S239

de Hann, Lieuwe, S378

de Jong, Steven, S238

de Luis-García, Rodrigo, S392

de Mamani, Amy Weisman, S265, S371

de Moura, Luciana Monteiro, S184

de Sousa, Ana Elisa Farias, S197

de Witte, Lot, S261

de la Foz, Victor Ortiz-García, S92, S184

de la Serna, Elena, S181, S183

de-Luis-Matilla, Antonia, S151

dos Santos, Antonio, S166

dos Santos, Bernardo, S371

dos Santos, Eriton, S378

Dhawan, Jasbeer, S65

Di Biase, Maria, S91

Di Carlo, Pasquale, S116

Di Fabio, Fabio, S393

Di Forti, Marta, S98, S110, S123, S158, S166, S354

Di Sarno, Elaine, S210

DiForti, Marta, S332

DiPetrillo, Lauren, S312

Diaconescu, Andreea, S254, S281, S314

Diaz, Camila, S364

Diaz-Caneja, Covadonga M., S226

Díaz, Luciana, S372

Dicker, Patrick, S74

Dickerson, Faith, S62, S63, S63, S87

Dickie, Erin, S176, S226

Dickson, Hannah, S143

Didriksen, Michael, S1

Dieset, Ingrid, S227

Dieter, Lesa, S374

Dietzek, Maren, S287

Diez, Alvaro, S186

Diez-Aja, Cristobal, S237

Dillon, Rachael, S349

Dipasquale, Ottavia, S83

Diukova, Anna, S283

Dixon, Lisa, S107

Dmitrienko, Alex, S236, S341

Do, Kim Q. Do, S395

Do, Kim Q., S2, S15, S133, S279, S310, S389

Do, Kim, S25, S25, S236

Docherty, Anna, S333

Dolan, Raymond, S385

Dollfus, Sonia, S390, S396

Dolmer, Signe, S134

Dolz, Montse, S229

Dolz, Montserrat, S183

Domènech, Cristina, S423

Dong, Fang, S241

Donkersgoed, Rozanne van, S238

Donohoe, Gary, S48, S141, S325, S325, S349

Doody, Gillian, S6, S77

Dooley, Niamh, S147, S181, S402

Dorner-Ciossek, Cornelia, S128, S380

Dotsch, Ron, S242

Douvold, Laëtitia Bouérat, S241

Downey, Linglee, S162, S270, S376

Downing, Donna, S54

Doyle, Joseph, S412

Draženka,Ostojić, S369

Drake, Richard, S50, S131, S148, S156, S197, S388

Drakesmith, Mark, S396

Drosos, Petros, S417

Drucaroff, Lucas, S286

Du Plessis, Stéfan, S367

Du, Xin, S338

Du, Ya-song, S333

Du, Yong, S8

Duan, Jubao, S34

Duarte, Rodrigo, S105

Dubath, Céline, S310

Dubovsky, Steve, S188, S189

Duburcq, Anne, S125

Dukart, Juergen, S83

Dulcis, Davide, S233

Dumais, Alexandre, S346

Duncan, Erica, S266

Duncan, Marcus, S413

Dunn, Graham, S131, S148

Dunn, Walter, S63

Dunne, Thomas Frederick, S280

Duval, Céline, S140

Dwyer, Dominic B., S185

Dwyer, Dominic, S100, S155, S246, S292, S316

Eap, Chin B., S236, S310

Eaton, Scott, S162, S270, S376

Eaton, William, S190

Ebbesen, Christine, S333

Ebdrup, Bjørn H., S289

Ebert, Dieter, S11

Edden, Richard, S20

Eduard, Bentea, S299 

Edwards, Jordan, S108, S271

Egan, Michael F., S85

Egerton, Alice, S51, S81, S89, S382

Egger, Stephan, S331

Eggers, Susanne, S285

Ehlers, René, S399

Ehrenreich, Hannelore, S85

Eichhorn, Peter, S261

Eickhoff, Simon B., S393

Eiroa-Orosa, Francisco José, 

Eisner, Emily, S197

Ek-Persson, Jane, S211

El Hechmi, Zouhaier, S163, S258

El Marroun, Hanan, S231

Eleftheriadis, Dina, S4

Elias, Beatriz Yukie, S375

Elise, Leroux, S390

Elkis, Hélio, S266

Elkis, Helio, S262, S371, S378

Ellersgaard, Ditte V., S110, S300

Ellersgaard, Ditte, S245, S252, S336

Elzinga, Bernet, S347

Emsley, Richard, S197

Emsley, Robin, S261, S364, S367

Ena,Ivezić, S257, S257

Engelstad, Katharina, S253

Engemann, Kristine, S59

English, Jane, S53, S72

Epdrup, Bjørn, S134

Eplov, Lene, S134

Ered, Arielle, S136

Erickson, Molly, S10

Ermiyev, Michael, S198

Ernala, Sindhu, S100

Erp, Theo van, S79

Escarti, Maria Jose, S424

Esen, Duygu, S316

Esmaeilzadeh, Abdolreza, S344

Espina-Eizaguirre, Alberto, S151

Espinosa, Justo Pinzón, S337

Espinosa, Laura, S337

Essig, Marco, S289

Esther Setién Suero, S259

Estrada, Francesc, S203, S251, S253, S303, S311

Ettinger, Ulrich, S119, S386

Eurelings-Bontekoe, Elisabeth, S341

Evans, Jonathan, S371

Evans-Lacko, Sara, S54, S55

Evenden, John, S248, S353

Everall, Ian, S74, S176, S224, S295

Evins, Anne Eden, S361

Ewertzon, Mats, S211

Fañanás, Lourdes, S330

Fabiana, Corsi-Zuelli, S222

Fachim, Helene, S329

Faerden, Ann, S369

Fagerlund, Birgitte, S80, S83, S213, S226, S232, S289

Faiola, Eliana, S119, S386

Faith, Laura, S362

Falkai, Peter, S17, S155, S261, S292, S293, S342, S343, S355, S393

Falkenberg, Irina, S195

Fallgatter, Andreas, S355

Fancis, Susan, S171

Fankam, Cédine, S17

Fanshawe, Thomas, S23

Faravelli, Laura, S126, S132

Farhall, John, S302

Farhat, sarah-Lise, S420

Farkash, Herman, S198

Farmah, Baldeep, S280

Fathian, Farivar, S324, S358

Fatjó-Vilas, Mar, S145, S174, S330, S397

Fatma Betül, Abut, S211, S419

Faulkner, Guy, S413

Fazel, Seena, S23

Fearon, Paul, S77

Fedechko, Taylor, S3

Federspiel, Andrea, S175, S184

Feher, Virginia, S192

Feixas, Guillem, S210

Feldman, Stephanie, S190

Felsenheimer, Anne, S136

Ferchiou, Aziz, S160, S166

Fernandes, Thalita, S146, S262, S349, S425

Fernandez-Castilla, Belén, S255

Fernandez-Egea, Emilio, S351, S360

Ferrara, Maria, S272, S426

Ferrari, Carina, S236, S389

Ferraro, Laura, S7, S75, S110, S158, S258, S354

Ferreira, Felipe, S255

Ferreira-Santos, Jair, S166

Ferretjans, Rodrigo, S22

Ferri, Mila, S426

Fervaha, Gagan, S267, S306, S309, S354, S393

Fethi, Guemira, S152

Figueiredo, Anna Suarez, S26

Filipova, Nina, S406

Fioramonte, Mariana, S53

Firmin, Ruth, S319

Firth, Joseph, S405

Fisher, Helen, S25, S97, S110, S118, S166

Fiszdon, Joanna, S196

FitzGerald, Thomas, S385

Fitzgerald, Zachary, S194

Fitzsimmons, Chantel, S296, S398

Flavia,Müller, S127, S234

Fleischer, Christian, S122, S228

Fleischhacker, Wolfgang, S50, S421

Fleischman, Anat, S23

Fletcher, Paul, S306

Flores, Cecillia, S37

Föcking, Melanie, S74

Foecking, Melanie, S53, S72

Foldager, Leslie, S252, S341

Foley, Fiona, S302

Fonseca, Ana Olívia, S255

Fonseca, Lais, S112

Fonseka, Trehani, S190

Fonville, Leon, S396

Forner, Mireia, S123

Fornito, Alexander, S91, S176

Forrest, Emma C., S132

Fortea, Adriana, S181, S183, S337

Forti, Marta Di, S7, S59, S75, S84, S246, S258, S364, S373

Foss-Feig, Jennifer, S266

Foster, Dan, S70

Foster, Daniel, S204

Fournier, Margot, S15, S25, S133, S236

Foussias, George, S176, S267, S306, S309, S354, S422

Fowler, David, S212

Fraccaro, Paolo, S106

Fraguas, David, S226

Frajo-Apor, Beatrice, S421

Francesco, Danilo Notar, S204, S312, S414

Francey, Shona, S162

Franchino-Elder, Jessica, S425

Francis, Andrew, S260

Francis, Emma, S84

Francis, Michael, S63

Franck, Nicolas, S47

Franck, Schürhoff, S166

Franco, Ana Luiza, S255

Frank, Michael, S340

Franscini, Maurizia, S230, S336

Fraser, Elizabeth, S118

Freeman, Daniel, S335

Freeman, Natalie, S218

Freitas, Elder Lanzani, S376

Freitas, Elder, S195

Freitas, Rosana, S262, S266, S371

Fresno, Andrés, S96

Freudenreich, Oliver, S361

Friedel, Evelyn, S11

Friedman, Zvika, S339

Friis, Svein, S272

Frisch, Katharina, S178

Fruchter, Eyal, S165

Fu, Susie, S250

Fuglø, Dan, S383

Fujimoto, Michiko, S419

Fujino, Haruo, S419

Fujito, Ryoshuke, S290

Funatogawa, Tomoyuki, S315

Funk, Adam, S19, S52

Furlan, Victoria, S210

Furnham, Adrian, S368

Furuichi, Atsushi, S394

Fusar-Poli, Paolo, S162, S323, S374

Fusar-poli, Paolo, S55, S56, S383

Fusaroli, Riccardo, S169, S350

Futatsubashi, Masami, S277

Gade, Katrin, S155

Gadelha, Ary, S96, S112, S153, S154, S161, S164, S184, S255, S266, S286, S375, S375

Gaebel, Wolfgang, S343

Gage, Suzanne, S165, S270

Gagné, Anne-Marie, S11, S124

Gago, Estefania, S251

Gagsteiger, Anna, S261

Gaisler-Salomon, Inna, S202

Gajšak, Tomislav, S257

Gajwan, Ruchika Gajwan, S385

Gajwani, Ruchika, S279, S282, S340, S409

Galbraith, Jordan, S204

Galderisi, Silvana, S50, S196, S263, S264

Gale, Tim, S220

Gallardo, Carlos, S269

Gallego, Carme, S174

Gallego, Juan, S221

Gallegos, Yuli, S123

Galletly, Cherrie, S73, S137, S208, S214, S284, S408

Galletti, Chiara, S201

Galli, Aurelio, S45

Galling, Britta, S108, S206

Gama, Clarissa, S22, S140, S288

Gamble, Clare, S178

Ganapathiraju, Madhavi, S189, S298

Ganella, Eleni, S91, S176

Gangadhar, B. N. , S136

Gantriis, Ditte L., S245, S300

Gantriis, Ditte Lou, S110, S336

Gantriis, Ditte, S252

Garcés, María Sol, S352

García-Bueno, Borja, S222

García-Mieres, Helena, S210, S317, S427

García-Rizo, Clemente, S332

Garcia, Cristina, S264, S353

Garcia, Jordi Soler, S330

Garcia, Miguel, S139

Garcia-Alvarez, Leticia, S119, S420

Garcia-Carmona, Juan-Antonio, S206

Garcia-Marti, Gracian, S276

Garcia-Portilla, M Paz, S119, S420

Garcia-Ribera, Carles, S410

Gardner-Sood, Poonam, S166

Garfinkel, Sarah, S173

Garrett, Chantel, S267

Garrido, Viviana, S96

Garverich, Suzanne, S137

Garzitto, Marco, S147

Gascoyne, Lauren, S171

Gaser, Christian, S122, S178, S287

Gass, Peter, S297

Gati, Joe, S179, S179

Gattaz, Wagner, S133, S195, S376

Gaughran, Fiona, S87, S166, S332

Gautier, Corinne, S420

Gawęda, Łukasz, S146, S252

Gawne, Timothy, S120

Gayer-Anderson, Charlotte, S7, S58, S110, S156, S158, S307, S354

Geaghan, Michael, S398, S400

Geels, Camilla, S167

Geerts, Hugo, S294, S411

Gehr, Johannes, S422

Gelao, Barbara, S100

Genberg, Maria, S407

Geng, Dawei, S69

Genske, Anna, S285

Geraets, Chris, S344, S419

Gerke, Wolfgang, S331

Gerretsen, Philip, S290, S329, S393

Gerstenberg, Miriam, S230, S336

Gertsik, Lev, S340

Gholam-Razaee, Mehdi, S236

Gholam-Rezaee, Mehdi, S310

Giacca, Adria, S104

Giampietro, Vincent, S154, S174, S413

Giannakopoulou, Alexandra, S243, S348

Giannakopoulou, Dimitra, S243, S348

Gicas, Kristina, S291

Giersch, Anne, S47

Gifford, George, S323

Gil, Roberto, S44

Gilbert, Elsa, S11, S142, S149, S230, S254

Gill, Kathryn, S1, S338

Gill, Michael, S357

Gillam, John, S316

Gilleen, James, S199

Gillespie, Amy, S382

Gillis, David, S61

Gilmore, John, S103, S194

Giménez, Anna, S337

Gingerich, Susan, S216, S320, S322

Gingras, Nathalie, S11

Giordano, Annalisa, S118, S131

Giordano, Giulia Maria, S264

Giordano, Giulia, S263

Girchenko, Polina, S26

Gisela, Sugranyes, S125

Gislum, Mette, S94

Gisselgård, Jens, S284

Giuliano, Anthony, S39

Gizdic, Alena, S196

Gjestad, Rolf, S324, S346, S358

Gladyshev, Igor, S213

Glass, Steven, S236, S341

Glatard, Anaïs, S310

Glausier, Jill , S29

Gleeson, John F, S4

Glenthøj, Birte Y., S289

Glenthøj, Birte, S80, S213, S383, S424

Glenthøj, Louise Birkedal, S119

Glenthoj, Birte, S50, S83, S119, S224, S226, S232, S314, S387, S422

Glerean, Enrico, S47

Glynn, Shirley, S216, S320, S322

Gobbicchi, Chiara, S363

Goedhart, Matthieu, S219

Goense, Jozien, S282

Gogate, Jagadish, S311

Goghari, Vina, S291, S327

Gohar, Sherif M., S227

Golay, Philippe, S236, S310, S395

Gold, James, S43, S140, S176, S190, S422

Goldman, Barbara, S103

Goldman, Howard, S4

Goldman, Robert, S239, S353

Goldring, Abraham, S244, S408

Goldstein, Benjamin I., S355

Goldzweig, Gil, S350

Gomes, Felipe, S21, S75

Gomes, Flavia C. A., S15

Gómez-Gastiasoro, Ainara, S347

Goncalves, Vanessa, S218

Gong, Qiyong, S92

González, Beatriz, S370

González, Fermin, S238, S348

González-Blanco, Leticia, S119, S420

González-Fernández, Alba, S251

González-Higueras, Fermín, S317

González-Rodríguez, Alexandre, S203, S311

Gonzales, Antonio, S312

Gonzalez, Alfonso, S269

Gonzalez, Carlos, S157, S200, S276

Gonzalez-Pinto, Ana, S222, S318

Gonzalez-Valderrama, Alfonso, S202, S364

Gooding, Diane, S198

Gooding, Patricia, S220

Goodyear, Bradley, S79

Gopal, Srihari, S130, S311

Gorbovskaya, Ilona, S190

Gordianno, Annalisa, S48

Gordon, Anne, S237

Gordon, Pedro, S133

Gore-Jones, Victoria, S237

Goulding, Anneli, S212, S426

Gowatsky, Jaimie, S331

Gowland, Penny, S180, S180

Goyal, Parth, S405

Gozlan, Guy, S125

Gräger, Stephanie, S287

Grace, Anthony, S1, S75, S338, S339, S416

Gracián, García-Martí, S384

Graff-Guerrero, Ariel, S290, S329, S354, S393

Grah, Majda, S257

Graham, Christine, S413

Graham, Stephen M, S132

Granger, Kiri, S145, S248, S353

Granholm, Eric, S77, S123, S195, S421

Grasa, Eva, S208, S210, S238, S317, S348, S372

Grases, Núria, S113, S212

Gray, David, S176

Grayson, Ben, S128

Grech, Adrienne, S338

Green, Alan, S235

Green, Mallik, S241

Green, Melissa, S229, S296, S347

Greene, Mallik, S97, S248, S366

Greenwood, Kathryn, S87, S212

Greenwood, Kathy, S173

Gregersen, Maja, S245, S300, S336

Greis, Kenneth, S52

Grent-’t-Jong, Tineke, S279, S282, S340, S385

Greve, Aja, S110, S245, S252, S300, S336

Griffa, Alessandra, S15, S25, S133, S279

Griffiths, Sian, S98

Grigis, Antoine, S172

Grigoriou, Markella, S159

Grinband, Jack, S331

Groc, Laurent, S60

Gronholm, Petra, S55

Groot, Christopher, S200

Gross, Amit, S202

Gross, Joachim, S279, S282, S340, S385, S409

Grottick, Andrew J., S128

Grove, Amber, S362

Gruber, Oliver, S275

Gruener, Daniel, S340

Gruetter, Rolf, S133, S236

Guardiola, Maria, S145, S174, S397

Gudbrandsen, Maria, S395

Guerrero-Pedraza, Amalia, S145

Guevara, Miguel, S172

Guevara, Pamela, S172

Guillory, Sylvia, S266

Guimaraes, Francisco Silveira, S28

Guimaraes, Francisco, S21

Guimond, Synthia, S121

Guinjoan, Salvador M., S286

Güliz,Özgen, S316

Guloksuz, Sinan, S84, S160, S272, S426

Guma, Elisa, S390

Gumley, Andrew, S279, S282, S385, S409

Gundersen, Steffie, S334

Gunther, Nicole, S160

Guo, Shuixia, S187

Gupta, Anoop, S405

Gupta, Tina, S42

Gur, Raquel, S57, S63

Gur, Ruben, S57, S63

Gurel, Seref Can, S345

Gurung, Bina, S405

Gurvich, Caroline, S137, S139, S143, S246, S252

Gussew, Alexander, S121, S221

Gutierrez-Zotes, Alfonso, S303

Güzide,Örüm, S316

Haaki, Willehard, S88

Haan, Lieuwe de, S99, S228

Haapea, Marianne, S144, S168

Haas, Shalaila, S168, S246

Haber, Lillian, S309

Haddock, Gill, S50

Haddock, Gillian, S220

Hafizi, Sina, S390

Häggmark-Månberg, Anna, S120

Hagen, Roger, S113

Hagi, Katsuhiko, S207, S363

Hagmann, Patric, S15, S25, S133, S279

Hahn, Margaret, S104, S190, S313, S413

Hahn, Peter, S30

Haidl, Theresa, S114, S209, S285

Hajak, Göran, S343

Hajdúk, Michal, S250, S360

Häkkinen, Katja, S88

Hakala, Mika, S94

Haker, Helene, S72, S281, S331

Hall, Emma, S171

Hall, Graham, S209

Hallak, Jaime Eduardo, S297

Hallett, Danielle, S334

Halpern, Brooke, S409

Haltigan, John, S422

Hamann, Stephan, S79

Hamilton, Emily, S85

Hamilton, Holly, S266

Hamilton, Matthew, S404

Hammerton, Gemma, S379

Hanen, Ben Ammar, S152

Hanna, Donncha, S317

Hanrahan, John, S72

Hansbauer, Maximilian, S343

Harada, Akina, S412

Hardingham, Kelton, S200

Hargovan, Hethal, S61

Hargreaves, April, S141, S349, S357

Haro, Josep Maria, S371

Harripaul, Ricardo, S296

Harris, Anthony, S192

Harris, Kamelia, S220

Harrisberger, Fabienne, S102, S199

Harte, Michael, S312

Hartley, Brigham, S34

Hartman, Catharina, S14

Hartman, Leah, S368

Hartman, Richard D., S132

Hartmann, Jessica A., S105, S124

Hartmann, Jessica, S378

Hartry, Ann, S97, S241, S248, S366

Harvey, Philip D., S353

Harvey, Philip, S40, S159, S208, S242, S320

Hasan, Alkomiet, S17, S261, S342, S343, S355, S393, S406, S416

Haselden, Morgan, S108

Hashimoto, Ryota, S85, S419

Hasking, Penelope, S379

Hasson-Ohayon, Ilanit, S260, S350

Hastrup, Lene Halling, S129

Hatada, Sanae, S290

Hathway, Pamela, S395

Haug, Elisabeth, S356, S410

Haugen, Ingvild, S356

Haugg, Amelie, S81

Hauke, Daniel, S102, S314

Hawar, Moradi, S159

Hawco, Colin, S176

Hawkins, Peter CT, S83

Hayata, Jacqueline, S18

Hayes, Richard, S411

Hazan, Hadar, S335

Hazlett, Erin, S240

He, Manxi, S331

He, Yujie, S82

He, Zhimin, S106

Healy, Colm, S109, S147, S181, S318

Heaney, Lisa, S312

Hébert, Marc, S11

Hechmi, Zouhaier EL, S371

Hechmi, Zouhaier El, S403, S423

Heffetz-Giterman, Lior, S202

Hefner, Amanda, S408

Hegelstad, Wenche ten Velden, S272

Heger, Anja, S128

Heidegger, Tonio, S340

Heilbronner, Urs, S155

Heinrichs, Walter, S149, S368

Heinsbroek, Jasper, S337

Heinz, Andreas, S172, S278

Heinze, Kareen, S209, S280, S370

Heinzle, Jakob, S314, S331

Heiskanen, Inkeri, S349

Helkis, Helio, S133

Helldin, Lars, S159, S263, S265

Hellemann, Gerhard H., S136

Hellström, Cecilia, S120

Hellvin, Tone, S113

Helm, Meghan, S188, S189

Hemager, Nicoline, S110, S245, S252, S300, S336

Henderson, David, S139

Hendriksen, Ingrid, S407

Henning, Bettina, S355

Henning, Draheim, S143

Henriksen, Maria Toft, S252

Henriques, Beatriz, S295

Herdener, Marcus, S284

Heretik, Anton, S250

Herman, Yarissa, S31

Hernandez, Miguel, S157, S424

Hernandez-Garcia, Maria, S157

Hernaus, Dennis, S182

Herold, Christina, S140, S289

Heron, Jon, S165, S270, S377, S379

Heslin, Margaret, S6

Hetrick, Sarah, S404

Heywood, Brodie, S295

Hickie, Ian B., S132

Hickie, Ian, S99, S116, S228, S378

Hickman, Matthew, S270

Hidalgo-Mazzei, Diego, S5

Hielscher, Emily, S379

Hietala, Jarmo, S69, S88, S272, S391

Higuchi, Cinthia, S153, S154, S161, S266, S375

Higueras, Fermin Gonzalez, S372

Hilker, Rikke, S80, S83, S213

Hill, Rachel, S338

Hillegers, Manon H.J., S231, S402

Hillegers, Manon, S77, S225, S287

Hinkley, Leighton, S278

Hirano, Yoji, S117

Hirjak, Dusan, S140, S289

Hirnstein, Marco, S261, S267, S302

Hitchcock, Steve, S339

Hjärthag, Fredrik, S159,S263, S265

Hjelmervik, Helene, S292

Hjorthøj, Carsten, S95, S111, S129, S163

Hjorthoej, Carsten, S365

Hjorthoj, Carsten, S99

Hjortshøj, Carsten, S134

Hlinka, Jaroslav, S173

Ho, Chun, S215

Ho, Newfei, S281

Ho, Wui Hang, S372

Hobson, Scott, S128

Hochard, Christophe, S420

Hodgins, Sheilagh, S143

Hoek, Hans W., S271

Hofer, Alex, S135, S421

Hoffman, Gabriel, S34

Hoffmeyer, Debra, S63

Hol, Elly, S82

Holland, Jessica, S325

Hollingworth, Sam, S178

Holmans, Peter, S379

Holzner, Bernhard, S135

Homan, Philipp, S330

Honer, William, S97, S290, S291, S343

Hong, Elliot, S20

Hong, Ji-Eun, S177, S411

Hong, Kyung Sue, S215, S362

Hong, L. Elliot, S327

Hong, Seok Jin, S47

Hong, Yeon-Ju, S308

Honkanen, Jarno, S68

Hood, Rebecca L, S20

Hopkins, David, S87

Hopkins, Richard, S106

Hopkins, Seth, S158

Hopkins, Stephen, S131, S148

Hopper, Shaun, S70

Hoptman, Matthew, S175, S331

Hopwood, Malcolm, S260

Horacek, Jiri, S173

Horacek, Magdalena, S128

Horga, Guillermo, S44

Hortêncio, Lucas, S195, S376

Houde, John, S278

Houenou, Josselin, S172

Houle, Sylvain, S43, S390

Houtepen, Lotte, S82

Howard, Robert, S377

Howes, Oliver, S13, S68, S81, S93, S225, S291, S330, S385, S395

Hsieh, Ming-Hsien, S186

Hsu, Jay, S114

Hsu, Yung-Chin, S186

Hu, Hui-xin, S358

Hua, Jun, S289

Hua, Lingling, S385

Huang, Debbie, S54

Huang, Jing-Ying, S186

Hubl, Daniela, S407

Huda, Samei, S156

Huerta-Ramos, Elena, S208

Hugdahl, Kenneth, S95, S113, S261, S292, S293, S302, S306

Hughes, Matthew, S78

Hughes, Rebecca, S233, S234

Huhtaniska, Sanna, S415

Hui, Christy, S97

Hui, Lai Ming, S138, S214, S215, S372

Hulkko, Anja, S144, S415

Hulshoff Pol, Hilleke E., S83

Hulshoff Pol, Hilleke, S82

Humberstone, Verity, S214

Humensky, Jennifer, S107

Hunnunen, Matti O., S379

Hurtig, Tuula, S101

Husain, Nusrat, S131, S148

Huynh, Helen, S415

Hwang, Na-Ri, S242

Hwang, Tae-Yeon, S240

Hwang, Tzung-Jeng, S186

Hwang, Wu Jeong, S294

Hwu, Hai-Gwo, S186

Hyötyläinen, Tuulia, S69

Hynninen-Sundelin, Ira, S345

Hyung, Lee Tak, S330

Hyytiäinen, Matias, S345

Iacoboni, Marco, S176

Iasevoli, Felice, S204, S312, S313, S414

Ibañez, Manuel Ignacio, S330

Ibad-Ramos, Marina, S167

Ibarretxe-Bilbao, Naroa, S347

Ichinose, Megan, S29, S77, S195, S196, S421

Idris, Nagi, S128

Iglesias, Celso, S119, S420

Iglesias, Sandra, S281, S331

Igor,Filipčić, S257, S257

Ik Cho, Kang, S186, S294

Ildiko, Piszar, S143

Ilvonen, Jenna, S345

Ilzarbe, Daniel, S125, S181, S183

Im, Chang-Hwan, S187

Immonen, Johanna, S144, S270

In Chan, Suet, S214

In Kang, Jee, S188

Incedere, Aysel, S419

Ingvild, Aase, S284

Inta, Dragos, S297

İncedere, Aysel, S211, S316

Iosif, Ana-Maria, S3, S126

Iqbal, Ehtesham, S411

Irani, Sarosh, S61

Iruretagoyena, Barbara, S202, S269, S364

Irvine, Karen, S220

Isdohanni, Matti, S168

Isenberg, Keith, S425

Ismail, Khalida, S87

Isohanni, Matti, S144, S270, S415

Isometsä, Erkki, S88

Isvoranu, Adela-Maria, S269, S367

Ithal, Dhruva, S136

Iversen, Trude S., S227

Iversen, Trude, S205, S362

Ivona Šimunović,Filipčić, S257, S257

Iwata, Yusuke, S290, S329, S393

Iyegbe, Conrad, S84, S156, S189, S332

Iyer, Srividya, S56

Jääskeläinen, Erika, S144, S168, S208, S270, S415

Jönsson, Erik, S205

Jørgensen, Hugo A, S346

Jørgensen, Hugo A., S358

Jørgensen, Robert, S153, S284

Jürgen,Dukart, S340

Jablensky, Assen, S214, S365

Jackowski, Andrea Parolin, S184, S286

Jackson, Richard, S154

Jacob, Arpitha, S181

Jacobs, Grace, S226

Jacobs, Jolien, S95

Jacobs, Nele, S84

Jaffe, Andrew E., S82, S85

Jaffee, Sara, S110

Jakobsen, Ane, S361

Jamain, Stephane, S51

James, Anthony, S229

James, Shaunequa, S322

Jang, Seon-Kyeong, S187, S260

Jansen, Philip R., S402

Janssen, Jaap, S183

Janssen, Ronald J., S101

Jauhar, Sameer, S13, S81, S395

Javadzadeh, Alborz, S221

Javitt, Daniel, S43, S331

Jeffrey Conn, P., S127, S204

Jenni, Raoul, S15, S25, S133, S236, S395

Jensen, Heidi Dorthe, S134

Jensen, Heidi, S95, S129

Jensen, Lars Thorbjørn, S383

Jensen, Maria H, S80

Jensen, Maria H., S83, S213

Jeon, Peter, S179, S179

Jeon, Youngsoon, S367

Jeong, Hyun-Ghang, S403

Jeong, Seong Hoon, S203

Jeppesen, Pia, S103, S163, S334

Jepsen, Jens Richardt Møllegaard, S226, S232, S245, S252,S300, S336

Jepsen, Jens Richardt, S111, S300

Jespersen, Cecilia Pihl, S103

Jessen, Frank, S285

Jessen, Kasper, S224, S289, S383, S387

Jessica, Graham, S162

Jewel, Charlotte, S208

Jha, Robin, S405

Jhon, Min, S411

Ji, Ellen, S172

Jiang, Wenlong, S329

Jiang, Ying, S312, S413

Jin, Huajie, S57

Jo, Anna, S411

Jo, Seongwoo, S367

Jo, Young Tak, S287

Joa, Inge, S153, S272, S284, S417

Joffe, Max, S127

Johannesen, Jason, S266

Johannessen, Jan Olav, S153, S272, S284, S417

Johansson, Anita, S211

Johansson, Daniel, S120

Johansson, Madeleine, S159, S265

Johansson, Ola, S211

Johansson, Sofia, S305

Johansson, Viktoria, S73

John, Majnu, S93

Johnsen, Erik, S95, S261, S292, S293, S324, S346, S358

Johnson-Kwochka, Annalee, S343

Jokela, Markus, S349

Jomphe, Valérie, S11, S124, S142, S149, S230

Jon, Duk-In, S161, S201, S206, S218, S264, S296, S304, S308, S367, S401, S411

Jones, Derek, S396

Jones, Hannah, S165, S270, S273, S379

Jones, Peter B., S6, S48, S379

Jones, Peter, S57, S58, S77, S131, S148

Jones-Brando, Lorraine, S150

Jongsma, Hannah, S58

Jönsson, Erik, S205

Joo, Eun-jeong, S397

Joo, Sung Woo, S287, S391

Joo, Yeon Ho, S215, S287

Joober, Ridha, S56, S148, S182, S185

Jørgensen, Hugo A, S346, S358

Jørgensen, Robert, S153, S284

Jürgen,Dukart, S340

Joutsenniemi, Kaisla, S88

Joyce, Dan, S164, S249, S375

Joyce, Eileen, S131, S148

Juárez, Alba Lubeiro, S145, S186, S205, S249, S392, S397

Jukuri, Tuomas, S88

Julio, Sanjuán, S384

Jung, Han-Yong, S257, S360

Jung, Hee Yeong, S203

Jung, Sra, S292

Jung, Wi Hoon, S386

Jung, Young-Hoon, S308

Just, David, S120, S223

Käkelä, Juha, S144

Kärgel, Christian, S128

Köhler-Forsberg, Ole, S116, S273

Kølbæk, Pernille, S342

Kübra,İpçi, S316

Kübra,Ipçi, S419

KIshimoto, Taishiro, S207

Kašpar, Ines, S257

Kadra, Giouliana, S411

Kahn, Moazzzam, S235

Kahn, René S., S51

Kahn, René, S50, S77, S82, S225, S261, S287

Kahn, Rene, S74, S247

Kaiser, Nathalie, S209

Kaiser, Stefan, S81, S284, S331

Kajanne, Risto, S88

Kakar, Rishi, S63

Kaladjian, Arthur, S381

Kalhovde, Anne Martha, S113

Kalivas, Peter W., S337

Kalman, Janos, S155

Kambeitz, Joseph, S114, S246, S283, S293

Kambeitz-Ilankovic, Lana, S147, S168, S246

Kameno, Yosuke, S277

Kaminski, Jakob, S172, S278

Kampman, Olli, S88

Kanagasundaram, Pruntha, S313

Kanazawa, Tetsu, S295

Kanba, Shigenobu, S117

Kandaswamy, Radhika, S25

Kane, John, S93, S100, S207, S216, S267, S320

Kang, Jihoon, S187

Kang, Jinsook, S240

Kang, Shi Hyun, S309

Kang, Yuree, S219

Kanji, Sarah, S190

Kao, Amy, S254

Karayiorgou, Maria, S399

Karelis, Antony, S17

Kariofillis, Daniela, S128

Karmacharya, Rakesh, S400

Karmacharya, Sarina, S184

Kasai, Kiyoto, S381

Kasparbauer, Anna, S119, S386

Katagiri, Naoyuki, S315

Kataoka, Yuzuru, S248

Kataria, Lakhan, S405

Katarina,Matić, S257

Kathuria, Annie, S400

Kato, Yasuhiko, S277

Katshu, Mohammad, S171

Katthagen, Teresa, S172, S278

Kaur, Amandeep, S129

Kaushal, Nidhi, S412

Kawasaki, Yasuhiro, S248

Kawohl, Wolfram, S331

Kebir, Oussama, S26

Keefe, Richard, S136, S159, S196, S281

Keeser, Daniel, S17, S406

Kegeles, Lawrence, S1, S2, S71

Keinänen, Jaakko, S68, S285

Kelleher, Eric, S141

Kelleher, Ian, S89, S100, S109, S147, S402

Kellendonk, Christoph, S44

Keller-Varady, Katriona, S17

Kelly, Allott, S162

Kelly, Deanna, S190

Kelly, Peter, S151

Kemmler, Georg, S135, S421

Kempton, Matthew, S307

Kennedy, James, S115, S218, S296, S399, S412

Kennedy, Sidney H., S355

Kenney, Joanne, S147

Kepinska, Adrianna, S189

Keshavan, Matcheri S., S36, S104, S121, S223, S366

Keshavan, Matcheri, S6, S14, S90, S194, S196, S236

Ketharanathan, Tharini, S224

Keysers, Christian, S89

Khadimallah, Ines, S25

Khan, Ali, S179, S179

Khan, Anzalee, S129, S159, S244, S275, S408

Khandaker, Golam, S48, S272, S325

Khanyaree, Ifrah, S246

Khelifa, Emira, S152, S163, S258, S371, S403

Kiang, Michael, S390, S415

Kidd, Martin, S364

Kidd, Sean, S31, S404

Kido, Mikio, S394

Kienzler, Anne-Kathrin, S61

Kieseppä, Tuula, S68, S80, S88, S172, S285, S345, S349

Kilian, Sanja, S261, S364, S367

Killackey, Eoin, S162, S214, S321, S334, S404

Killen, Jefferey, S120

Kilpatrick, Gavin, S339

Kim, Do-Won, S187

Kim, Edward, S130, S311

Kim, Euitae, S382, S386

Kim, Eun Jin, S344

Kim, Han-Suk, S307

Kim, Harin, S287

Kim, Hyo-Won, S232

Kim, Jae-Jin, S308

Kim, Jae-Kyeong, S177

Kim, Jae-Min, S177, S411

Kim, Jeong Hoon, S292, S293

Kim, Julia, S290, S329, S393

Kim, Jung Jin, S177

Kim, Kyung Ran, S188

Kim, Min-Woo, S308

Kim, Minah, S186, S203

Kim, Moon Doo, S161, S201, S215, S236, S296, S304, S308, S326, S411

Kim, MoonDoo, S206, S401

Kim, Pitna, S299

Kim, Se Hyun, S203

Kim, Se Joo, S188

Kim, Seoyoung, S386

Kim, Seung Hyun, S403

Kim, Soo-Jeong, S308

Kim, Sun-Min, S240

Kim, Sung-Wan, S177, S411

Kim, Sungkean, S187

Kim, Tae Yong, S257

Kim, Yong Sik, S203

Kimhy, David, S17, S233

Kimura, Haruhide, S412

Kimura, Kohei, S248

King, Daniel, S208

Kiras, Fatma, S211, S283, S316, S419

Kircher, Tilo, S276

Kirkbride, James, S58, S377

Kirschner, Matthias, S81, S174, S284

Kirstie-Kulsa, Mila, S129

Kisely, Steve, S66, S178

Kjelby, Eirik, S261

Klaasen, Nicky, S177

Klauser, Paul, S15, S25, S133, S279

Klein, Hans, S358, S360

Kleinman, Leah, S241

Kleinmann, Joel E., S82

Klim, Casimir, S43, S331

Klingberg, Stefan, S128

Klingenberg, Boris, S84

Knable, Michael, S274, S404

Knegtering, Henderikus, S177

Knevel, M.F., S256

Knox, Emma, S148

Knudsen, Christina Bruun, S336

Ko, Mi-Ae, S267

Koblan, Kenneth S., S158

Koenig, Thomas, S263, S264

Koevoets, Martijn, S225

Kohler, Christian G., S305

Kohler, Christian, S57

Koike, Shinsuke, S215, S381

Kokkinou, Michelle, S93

Kokoszka, Andrzej, S252

Kolliakou, Anna, S166

Kometer, Michael, S281

Kompus, Kristiina, S113, S261, S267, S292

Kondofersky, Ivan, S155

Kondziella, Daniel, S116

Kontis, Dimitrios, S243, S348

Kopala, Lili, S291

Koponen, Hannu, S144

Koppel, Alex, S390

Koren, Dan, S54, S198, S306, S324

Korenic, Stephanie, S20

Korpela, Hanna, S144

Kos, Claire, S177

Kosidou, Kyriaki, S67

Kothare, Hardik, S278

Kotov, Roman, S6, S40

Kott, Alan, S131, S135

Kotz, Sonja, S380

Koutsouleris, Nikolaos, S100, S114, S119, S144, S147, S155, S168, S185, S209, S246, S283, S292, S293, S316, S328, S386

Kowalchuk, Chantel, S104, S313

Kozakova, Eva, S173

Kozauer, Susan, S236, S341

Kråkvik, Bodil, S113

Krężołek, Martyna, S146, S252

Kraguljac, Nina, S41, S91

Kravitz, Richard, S4

Krebs, Marie-Odile, S26, S268, S371

Krishnan, Ranga, S281

Kristensen, Jette Kolding, S88

Kristensen, Tina Dam, S119

Krivoy, Amir, S339

Krogh, Jesper, S116

Kroken, Rune A., S95, S292

Kroken, Rune, S324, S346, S358, S417

Kruiper, Caitlyn, S314

Krynicki, Carl, S48, S131

Krystal, John, S1

Ku, Benson, S167

Kubicki, Marek, S184, S194

Kubli, Roman, S284

Kudo, Noriko, S419

Kuhnt, Katrin, S122, S228

Kulkarni, Jayashri, S214

Kumar, Devvarta, S345

Kumar, Divya, S3

Kumar, Jyothika, S171

Kumari, Veena, S89

Kundu, Prantik, S240

Kunze, Birgit, S343

Kurdyak, Paul, S108, S271

Kurzthaler, Ilse, S135

Kushner, Steven A., S231, S402

Kusztrits, Isabella, S261, S267, S302

Kwak, Yoo Bin, S294

Kwiatkowski, Molly, S233

Kwon, Jun Soo, S186, S203, S215, S294, S386

Kwon, Young Joon, S201, S218, S236, S326, S411

Kwon, Young-Joon, S206, S215, S308, S401

Kyoon An, Suk, S188

Kyung-jin, Lho, S330

kian, Alireza Armani, S344

Lähteenvuo, Markku, S88

Lässer, Marc, S140, S289

López-Carrilero, Raquel, S317, S372

López-Vílchez, Irene, S332

Lönnqvist, Jouko, S88

Løberg, Else-Marie, S292, S324, S346, S358

La Barbera, Daniele, S354

La Cascia, Caterina, S110, S354

Labad, Javier, S203, S251, S253, S303, S311, S370

Labouesse, Marie, S65

Lacerda, Acioly, S133

Lacoua, Liza, S198

Lada,Goršić, S257

Ladea, Maria, S160

Lagerberg, Trine V., S113, S362

Lahti, Adrienne, S41, S91, S120

Lahti, Jari, S26

Lally, John, S81, S87, S332, S373

Lalousis, Paris Alexandros, S280, S283

Laloyaux, Julien, S113, S261, S267

LaMantia, Anthony, S1

Lambert, Thierry, S420

Lanari, Marcello, S123

Lancaster, Tom, S30

Land, Rachel, S66

Lander, Sharon S., S202

Landgrebe, Michael, S343

Lang, Donna, S291

Lang, Undine E., S199

Langbein, Kerstin, S121, S122, S221, S228, S287

Langguth, Berthold, S342, S343

Lappin, Julia, S6

Laprevote, Vincent, S169

Larøi, Frank, S113, S261, S267

Lara, Baldwin, S162

Larnaout, Amine, S152, S163, S258, S371, S403, S423

Larsen, Tor Ketil, S417

Larsson, Henrik, S23

Laskaris, Liliana, S74

Lattanzi, Guido Maria, S393

Lau, Cindy, S108, S271

Lau, Fu Chun, S215

Laughlin, Christopher, S218

Laurens, Kristin R, S118

Laurens, Kristin R., S229

Laurens, Kristin, S55, S143

Laurikainen, Heikki, S391

Lauritsen, Marlene Briciet, S333

Laursen, Thomas Munk, S87, S88, S111

Lavie, Adi, S350

Lavoie, Suzie, S326, S378

Lawire, Stephen, S385

Lawrence, Richard, S359

Lawrie, Stephen, S131, S148, S279, S282, S340, S409

Laws, Keith, S220

Leacy, Finbarr, S379

Lebel, Catherine, S355

Leboyer, Marion, S51, S160, S166, S172

Lecardeur, Laurent, S125

Lee, Arira, S292, S293

Lee, Chewah, S176

Lee, Edwin, S97

Lee, Erin, S247

Lee, Eun, S188

Lee, Eunbyeol, S242

Lee, Ho Ming, S138, S214, S215, S372

Lee, Hoi Ching, S138, S214

Lee, Hoseon, S360

Lee, I Hui, S389

Lee, Jimmy, S123, S270, S281, S309, S324

Lee, Jong Il, S309

Lee, Ju-Yeon, S177, S411

Lee, Jung Suk, S267

Lee, Jung Sun, S219, S232, S287, S387, S391

Lee, Junhee, S186, S203

Lee, Kang-Soo, S293

Lee, Kwanghun, S161, S201, S215, S236, S264, S296, S326, S367, S411

Lee, Kyuyoung, S397

Lee, Nam Young, S203

Lee, Sang Yup, S215

Lee, Sang-Hyuk, S292, S293

Lee, Sang-Yeol, S161, S201, S215, S218, S236, S264, S296, S304, S308, S367, S411

Lee, Seon-Koo, S267

Lee, Seung-Hwan, S187, S240

Lee, Stuart, S321, S359

Lee, Su Young, S188

Lee, Sun Woo, S293

Lee, Tae Young, S186, S203, S294

Lee, Tatia M.C., S243

Lee, Won-Hye, S240

Lee, Yu Sang, S215

Legind, Christian, S80, S83, S213

Lei, Wan U, S95

Leiponen, Marita, S345

Leite, Isabel, S61

Lemvigh, Cecilie, S232

Lenartowicz, Ewelina, S65, S191

Lencz, Todd, S221, S330

Lenderking, William, S241

Lennox, Belinda, S60, S61, S254

Lepage, Martin, S56, S148, S182, S185, S197

Leroux, Elise, S396

Lesh, Tyler, S4, S126

Letendre, Elaine, S17

Leucht, Claudia, S94

Leucht, Stefan, S51, S94, S94, S115, S305, S342, S418

Leung, Patrick, S161

Levier, Axel, S310

Lewis, Cathryn, S7, S158

Lewis, Glyn, S39, S48, S53, S72, S165, S247, S270, S379

Lewis, Shon, S50, S106, S131, S148, S156

Leza, Juan Carlos, S222

Li, Anna, S188, S189

Li, Chunbo, S36, S366

Li, Fangyong, S272

Li, Gang, S103

Li, HuiJun, S36, S366

Li, Huijun, S90, S125, S218

Li, Mingli, S79, S82, S90, S394

Li, Tao, S79, S82, S90, S121, S301, S394

Li, Xianbin, S329

Li, Xiaojing, S90, S394

Li, Yinfei, S90, S394

Li, Zhi, S382

Liang, Yumin, S11

Libas, Yulia, S54

Librenjak, Dina, S257

Lichtenstein, Thorsten, S114

Liddle, Elizabeth, S171

Liddle, Peter, S170, S170, S171, S171, S180, S180, S180, S283

Lieberman, Jeffrey, S115, S218

Light, Gregory, S266

Liharska, Lora, S159

Lim, Carmen, S237

Lim, Eunsung, S201, S206, S215, S218, S304, S326, S367, S401, S411

Lim, Issel, S289

Lim, Joseph K.W., S281

Lim, Steffiany, S323

Lima, Murilo, S262

Lin, Ashleigh, S209, S334, S370

Lin, Bochao, S84

Lin, Yi-Tin, S186

Lincoln, Alisa, S137

Lincoln, Tania M., S410

Lindberg, Nina, S88

Lindemann, Lothar, S1

Linden, David EJ., S379

Linden, David, S30, S396

Lindenmayer, Jean-Pierre, S129, S175, S244, S275, S408

Lindgren, Maija, S285, S349

Lindsley, Craig, S70, S127, S204

Link, Bruce, S54

Linscott, Richard J., S229

Linscott, Richard, S150, S256, S335, S351, S399

Lisiecka, Danuta, S131, S148

Little, Simon, S416

Liu, Bangyan, S400

Liu, Chen-Chung, S186, S274, S414

Liu, Chenxing, S282, S295

Liu, Chih-Min, S186

Liu, Dennis, S73, S137, S208, S284

Liu, Fang, S190

Liu, Lu, S231

Liu, Siwei, S281

Liu, Tsz Ting, S215

Liu, Wen-hua, S244

Ljuri, Isidora, S129, S244, S275

Llacer, Blanca, S424

Llorca, Bibiana Cabrera, S222, S318

Lo, Cecilia, S298

Loas, Gwenole, S363

Lobo, Antonio, S222

Loch, Alexandre Andrade, S195

Loch, Alexandre, S376

Lockwood, Jonathan, S413

Lodge, Daniel, S101

Lodhi, Rohit, S295

Loebel, Antony, S114, S158, S239, S242, S353

Loewy, Rachel, S4, S113

Lopez, Daisy, S371

Lopez, Lorna, S53, S72

Lopez-Carrilero, Raquel, S238, S348

Lopez-Morinigo, Javier-David, S166, S374

Lorente, Esther, S200, S238, S348, S372

Lorenzo, Giorgio Di, S263, S264

Loureiro, Camila, S166, S227, S329

Lourenço, Joca Sâmia Regiane, S222

Loures Malinowski, Fernando Rocha, S161

Louzã Neto, Mario Rodrigues, S210

Louzada, Paulo, Jr., S329

Louzada-Junior, Paulo, S166, S227

Louzada-Junir, Paulo, S222

Lovko, Sandra Kocijan, S257

Lu, Wei-hong, S358

Ľubica,Forgáčová, S250

Lui, Simon S.Y., S333, S358

Lui, Su, S331

Luis, Martí-Bonmatí, S384

Luk, Sin Ki, S243

Lunden, Anya, S409

Lunding, Synve Hoffart, S362

Luo, Xue-rong, S333

Luoni, Alessia, S297

Lussier, Daphné, S11, S124, S142, S230

Luteijn, L.B., S256

Luther, Lauren, S319, S343, S350

Luykx, Jurjen, S84, S261

Lyall, Amanda, S194

Lyngstad, Siv Hege, S369

Lynn, Marquardt, S302

Lynskey, Michael, S259

Lysaker, Paul, S238, S260, S319, S350

Lystad, June U., S113

Lythgoe, David J., S81, S89

Mäki, Pirjo, S101, S164

Männynsalo, Teemu, S88

Mäntylä, Teemu, S80, S285

Månberg, Anna, S120, S223

Möller, Julian, S331

Møllegaard Jepsen, Jens Richardt, S111

Møller, Paul, S410

Mørch, Ragni H., S205, S227

Mørch, Ragni Helene, S362

Ma, Xiaohong, S90, S394

MacCabe, James, S66, S67, S81, S143, S382

MacDonald, Angus,III S140

MacDougall, Arlene, S108, S271

MacDowell, Karina, S222

MacEwan, William, S291

MacKenzie, Erin, S133

MacKenzie, Malgorzata, S190

MacQueen, Glenda, S355

Macaluso, Matthew, S63

Machin, Matt, S106

Macht, Deliah, S250

Maciukiewicz, Malgorzata, S218

Mackenzie, Lynn, S252

Mackinley, Michael, S179, S179

Mackinnon, Andrew, S378

Maclaurin, Sarah, S361

Maddalena, Giancarlo, S85

Maddock, Richard, S126

Madonia, Jennifer, S176

Magata, Yasuhiro, S277

Maguire, Áine, S357

Maguire, Gerald, S63

Mahfouda, Simone, S334

Mahmoudi, Ebrahim, S398

Maihofer, Adam, S82

Maj, Mario, S196

Makowski, Carolina, S182, S185

Makris, Nikos, S184

Makuch, Mateusz, S61

Malchow, Berend, S17, S152, S343

Malhotra, Anil, S93, S176, S221, S330, S422

Malla, Ashok, S56, S148, S182, S185, S371

Mallar Chakravarty, M., S121, S290

Mallett, Susan, S23

Mallikarjun, Pavan, S280, S283, S328

Mamo, David, S393

Mancilla, Felipe, S269

Mandl, Rene C. W., S109, S289

Mandl, Rene, S83

Mane, Anna, S237

Manoliu, Andrei, S81, S284

Manseau, Marc, S167, S201

Mansueto, Giovanni, S76

Mantere, Outi, S68, S285

Mantyla, Teemu, S172

Mao, Xiangling, S2

Maples, Natalie, S31, S31

Mar, Wanna, S393

Marcó-García, Silvia, S208

Marcelino, Loureiro Camila, S222

Marcelis, Machteld, S67, S117

Marcus, Chiu Yu-Lung, S427

Marder, Stephen, S63

Marek, Kenneth, S176

Marenco, Stefano, S85

Mareschal, Isabelle, S249

Margolis, Russell, S289

Mario, Alvarez-Jimenez, S162

Markulev, Connie, S99, S116, S132, S228, S326, S378

Markus, Schwab, S380

Marmo, Federica, S313

Marquardt, Lynn, S261

Marques, Tiago Reis, S77, S92, S98, S246, S291

Marquet, Pierre, S230, S254

Marroun, Hanan El, S402

Marshall, Louise, S416

Marshall, Max, S156

Marshe, Victoria, S190

Marsman, Jan Bernard, S177

Marta, Del-Ben Cristina, S222, S227

Marteleur, Alexander, S407

Marti-Bonmati, Luis, S276

Martin, Brice, S47

Martin, Graham, S379

Martin, Nicholas, S12

Martin-Iverson, Mat, S365

Martinez-Aran, Anabel, S123

Martins, Diana, S262

Martins, Thaís, S146, S262

Martins, Thais, S349, S425

Martins-De-Souza, Daniel, S15, S22, S41, S51, S53, S297

Marvulli, Daniela, S116

Marwaha, Steven, S48

Marzi, Sarah, S25

Marzullo, Giovanni, S193

Masana, Guillem, S332

Masellis, Rita, S100, S193

Mashhadi, Farzaneh, S368

Massie, Ann, S20

Massons, Carmina, S370, S410, S423

Massuda, Raffael, S288

Massuda, Raphael, S2

Mates, Sharon, S236, S341

Mateu, Ainoa, S5

Mathalon, Daniel H., S78, S231

Mathalon, Daniel, S62, S79, S102, S266, S396, S422

Mathews, Maju, S130, S311

Mathys, Christoph, S278, S281

Matrone, Marta, S312

Matsuda, Tokio, S394

Matsumoto, Hiroshi, S315

Matsuoka, Jun, S381

Mattaroccia, Silvia, S95

Mattila, Ismo, S69

Mawn, Lauren, S321

Mayer, Jutta, S250

Maynard, Thomas, S2

Maziade, Michel, S10, S11, S124, S142, S149, S230

Mazloomzadeh, Saeedeh, S344

McAlonan, Grainne, S183

McCLure, Margaret, S240

McCarley, Robert W., S117, S223, S366

McCarley, Robert, S90

McCarthy, Timothy, S176

McCartney, Lara, S66

McCrory, Eamon, S331

McCullumsmith, Robert E., S299

McCullumsmith, Robert, S19, S19, S52

McCutcheon, Rob, S225

McDaid, David, S59

McDonald, Mhairi, S409

McDonnell, David, S312, S413

McEwen, Sarah, S18, S79, S396

McFarlane, William, S54

McGavin, Jacob, S153

McGlashan, Thomas H., S78, S102, S231

McGlashan, Thomas, S62, S79, S266, S396

McGorry, Patrick D, S74

McGorry, Patrick D., S4, S105, S116, S124, S132, S162, S228, S270, S326

McGorry, Patrick, S99, S130, S162, S214, S376, S378

McGrath, John, S87, S167, S270

McGuire, Philip, S27, S28, S51, S56, S74, S81, S81, S83, S118, S154, S162, S174, S307, S310, S323, S328, S374, S383, S393, S413

McGurk, Susan, S244, S404

McIlwaine, Sarah, S56

McIntyre, Gina, S295

McKenna, Peter J., S174

McMillan, Greg, S216

McMullen, Katrina, S89

McNulty, James, S150

McQueen, Grant, S81, S382

Mcglashan, Thomas, S39

Meador-Woodruff, James, S52, S298, S299, S400

Mechelli, Andrea, S92, S331, S374

Medalia, Alice, S196

Medeiros, Heydrich, S262

Meecham, Sabrina, S283

Meehan, Stine R., S241

Meehan, Stine, S94, S133

Mehdyoun, Nicole, S63

Mehl, Stephanie, S410

Mehmet Buğrahan, Gürcan, S316

Mehtälä, Tuukka, S349

Mehta, Mitul A., S118

Mehta, Mitul, S83

Mehtala, Tuukka, S32

Meier, Madeline, S12

Meisenzahl, Eva, S144, S147, S209, S328

Meisner, Christoph, S128

Melau, Marianne, S95, S106, S129

Melle, Ingrid, S113, S153, S205, S227, S272, S362, S369, S410

Meller, Jarek, S52, S299

Mellgren, Zophia, S211

Meltzer, Herbert, S115, S218

Mena, Cristian, S202, S364

Mendes, Diego, S146, S262, S349, S425

Mendyk, Nina, S198

Menezes, Paulo Rossi, S329

Menezes, Paulo, S166

Meng, Ya-jing, S90, S394

Menne-Lothmann, Claudia, S84

Meola, Annasara, S421

Mercedes Perez-Rodriguez, M., S240

Mercer, Jeremy, S284

Mercuel, Alain, S167

Merette, Chantal, S149

Merlino, Sergio, S162

Merriman, Tony, S399

Mesa, Nora, S251

Mesholam-Gately, Raquelle, S194

Meshul, Charles K, S20

Mesman, Esther, S225

Metzak, Paul, S388

Metzler, Sibylle, S230, S336

Meunier-Cussac, Sophie, S125

Meyer, Denny, S139, S302

Meyer, Eric, S39

Meyer, Monet, S4

Meyer, Urs, S2, S8, S65, S234

Meyer-Kalos, Piper, S216, S320, S322

Meyer-Lindenberg, Andreas, S51

Meyhöfer, Inga, S119, S386

Mezquida, Gisela, S222, S318, S351

Michael Deans, PJ, S105

Michel, Chantal, S109, S228, S407

Mielnik, Catharine, S19

Miettunen, Jouko, S144, S168, S270, S415

Miguel, Euripedes C., S286

Mikanmaa, Emmi, S279, S282, S385

Mike, Luke, S121

Mikolajacks, Gladys, S17

Milak, Matthew, S2

Mill, Jonathan, S25

Miller, Brian, S242

Millman, Zachary, S43

Milovac, Željko, S257

Mimica, Nino, S257

Mimura, Masaru, S329

Minah, Kim, S330

Miñano, Maria José, S303

Mingoia, Gianluca, S169, S277, S380

Minichino, Amedeo, S162

Minyaycheva, Maria, S213

Miselli, Manuela, S426

Misheneva, Varvara, S150

Mitelman, Serge, S268

Mitjans, Marina, S85

Mitra, Sayantanava, S136

Mittal, Vijay, S42

Mizrahi, Romina, S43, S309, S390

Mizuiri, Danielle, S278

Mizuno, Masafumi, S315

Modinos, Gemma, S89

Moehle, Mark, S204

Moeller, Scott, S240

Moffitt, Terrie, S25, S97, S110

Mofsen, Rick, S340

Moghaddam, Bita, S45

Mohn, Christine, S141, S245, S401

Moilanen, Jani, S144, S415

Moisés, Ana Carolina, S226

Mok, Pearl L.H., S167

Molden, Espen, S205

Molero, Patricio, S352

Molina, Vicente, S186

Mollon, Josephine, S39, S77, S247

Molteni, Raffaella, S202

Mondelli, Valeria, S118, S118, S166, S246

Monenschein, Holger, S339

Mongan, David, S317

Monreal, José Antonio, S203, S251, S253, S303, S311

Monserrat, Clara, S410

Montalvo, Itziar, S253, S303, S311

Moore, Cynthia, S20

Moore, Tyler M., S305

Moore, Tyler, S57

Moradi, Hawar, S263, S265

Moreira, Fabricio, S21

Moreno, Daniel, S237

Moreno, Marcos, S174

Moreno-Kustner, Berta, S151

Moretti, Patrizia, S363

Morgan, Craig, S6, S7, S58, S58, S59, S77, S110, S156, S158, S166, S166, S307, S354

Morgan, Kevin, S77, S368

Morgan, Vera, S365

Morishita, Hirofumi, S2

Moritz, Rossner, S380

Morkved, Nina, S324

Morley, Thomas, S176

Morris, Brian, S234

Morris, Crystal, S216

Morris, Derek, S325

Morris, Peter, S171

Morrison, Paul, S27

Morrison, Sinead, S86

Mors, Ole, S111, S134, S163, S245, S252, S300, S336, S341, S342, S350

Mortensen, Erik Lykke, S252

Mortensen, Preben Bo, S59, S87

Mortensen, Preben, S85, S167

Mossaheb, Nilufar, S99, S116, S132, S228, S378

Mothersill, David, S141

Mouchlianitis, Elias, S92, S383

Mougin, Olivier, S180

Moya, Jorge, S330

Mucci, Armida, S50, S196, S263, S264

Muckenhuber-Sternbauer, Susanna, S292

Mueller, Daniel, S115, S190, S218, S239, S412

Mueller, Nikola, S155

Mueller, Toni, S298

Mueser, Kim, S216, S320

Mulert, Christoph, S9

Mulholland, Ciaran, S317

Mullier, Emeline, S279

Mulligan, Victoria, S339

Mulsant, Benoit, S176, S393

Munafò, Marcus, S165

Munafo, Marcus, S270

Munday, Jennifer, S97, S366

Munk-Olsen, Trine, S88

Muñoz, Daniel, S113

Muralidharan, Anjana, S40

Murphy, Timothy, S288

Murphy, Veronica, S103

Murray, Graham, S101, S144, S164

Murray, Robin M., S166

Murray, Robin, S6, S7, S58, S59, S75, S77, S84, S87, S92, S98, S110, S118, S123, S156, S158, S166, S189, S246, S258, S328, S332, S354, S364, S373

Muscat, Whitney, S267

Mussa, Amjad, S324

Mustafé, Gustavo, S146, S262, S349, S425

Mustafa, Sally, S56

Myers, Neely, S178

Myin-Germeys, Inez, S307

Myles, Hannah, S284

Myles, Nicholas, S284

Nagarajan, Srikantan, S278

Nagele, Robert, S328

Nagels, Arne, S195, S276

Najdenovska, Elena, S395

Nakaizumi, Kyoko, S277

Nakajima, Shinichiro, S290, S329, S393

Nakamura, Mihoko, S394

Nakatani, Atsushi, S412

Nam, Beomboo, S308

Nam, Beomwoo, S161, S206, S215, S264, S296, S326, S367, S401

Napolitano, Isabel Cristina, S210

Napolitano, Isabel, S262

Narayanaswamy, Janardhanan C., S345

Nascimento, Juliana, S15

Natesan, Sridhar, S65, S191

Naveen Kumar, C., S136

Nawani, Hema, S345

Nefzi, Rahma, S152, S163, S258, S371, S403, S423

Neill, Erica, S139, S252, S355

Neill, Joanna, S128, S312

Nelson, Barnaby, S4, S46, S74, S99, S105, S116, S124, S132, S162, S228, S326, S370, S378, S410

Nemoto, Kiyotaka, S248

Nemoto, Takahiro, S315

Nenad,Jakšić, S257

Nenadic, Igor, S169, S178, S287, S380

Nentwich, Beate, S128

Netser, Roni, S10, S401

Neuchterlein, Keith, S63

Neumann, Suzanne, S351

Nevado, Angel, S186

Neves, Gilda, S416

New, Antonia, S240

Newcomer, John W., S114

Newman, Ellie, S237

Ng, Boon Tat, S309

Ni, Hua, S358

Ni, Peiyan, S121, S301

Nichols, Heathman, S195, S196, S421

Nicholson, Timothy, S328

Nick, Medford, S173

Nicolas, Poirel, S390

Nielsen, Anita Tønder, S88

Nielsen, Mai, S134

Nielsen, Mette Ø., S289

Nielsen, Mette Ødegaard, S224, S383, S424

Nielsen, Mette Odegård, S387

Nielsen, Mette, S80, S213, S422

Nielsen, René Ernst, S333

Nielssen, Olav, S214

Nieman, Dorien H., S132, S228

Nieman, Dorien, S99, S116, S378

Niemi-Pynttäri, Jussi, S88

Niendam, Tara, S3, S4

Nieto, Lourdes, S370, S410, S423

Nievergelt, Caroline, S82

Nijman, Henk, S24

Nijman, Saskia, S419

Nikita, Setiaman, S225

Niklas, Schülert, S380

Nikolina Tunjić,Vukadinović, S257

Nilsson, Hanna, S120

Nilsson, Peter, S120, S223

Nilsson, Sandra Feodor, S111

Nimgaonkar, Vishwajit, S63

Niolu, Cinzia, S263, S264

Nishikawa, Yumiko, S394

Nissen, Wiebke, S380

Niswender, Colleen, S127

Niznikiewicz, Margaret A., S117

Niznikiewicz, Margaret, S36, S90, S223, S266, S366

Noethen, Markus M., S287

Noguchi, Kyo, S394

Norbeck, Oscar, S120

Nordentoft, Merete, S50, S85, S95, S99, S99, S106, S110, S111, S116, S116, S119, S129, S132, S134, S163, S213, S228, S245, S252, S273, S300, S300, S336, S365, S378

Nordström, Tanja, S144

Nordstroem, Anna-Lena, S371

Norman, Ross, S108, S207, S271

Norton, Joanna, S359

Nosaka, Tadashi, S363

Nossel, Ilana, S107

Noto, Cristiano, S96, S153, S154, S161, S184, S255, S266, S375, S375

Noto, Mariane, S96

Notter, Tina, S8

Nour, Matthew, S81, S385

Noviar, Galoeh Adrian, S157

Nuamah, Isaac, S130

Nudel, Ron, S85

Nuechterlein, Keith H., S136

Nuechterlein, Keith, S16, S18

Nummenmaa, Lauri, S47, S77

Nunez de Arco, Rossana Kathenca Peredo, S149

Nunez, Daniel, S96

Nurmi, Erika, S218

Nye, Kathleen, S3

O’ Hanlon, Erik, S147

O’Connor, Kieron, S346

O’Daly, Owen, S174, S280, S291

O’Donnell, Colin, S130

O’Donnell, Lauren J., S194

O’Donnell, Maryanne, S73

O’Donnell, Patricio, S2

O’Donoghue, Brian, S162, S270, S376

O’Donovan, Michael C., S273, S379

O’Donovan, Michael, S7, S165

O’Donovan, Sinead, S19

O’Gorman, Aoife, S53, S72

O’Hanlon, Erik, S89, S181, S402

O’Hare, Kirstie, S399

O’Leary, Conor, S37

O’Neill, Aisling, S154, S384

O’Reilly, Nicole, S347

Ochoa, Susana, S151, S208, S210, S212, S238, S317, S348, S370, S372, S410

Ochoa, Susana, S151, S208, S210, S212, S238, S317, S348, S370, S372, S410

Odejayi, Gbolahan, S418

Oduola, Sherifat, S58

Ofray, Dayshalis, S142

Oh, Hyeonju, S260

Oh, Ji-Hye, S242

Ohi, Kazutaka, S248, S419

Ohmann, Christian, S343

Ohrablo, Peter, S250

Øie, Merete, S356, S410

Ojeda, Natalia, S347

Ojeil, Najate, S2

Okada, Naohiro, S381

Okubo, Hiroaki, S248

Olasz, Irina, S251

Olesen, Julie Aamand, S365

Olga, Soprano-Ros, S384

Oliveira, Graça, S262

Oliveira, Hianna, S154

Oliver, Dominic, S162, S323

Oliver, Lindsay, S422

Olivier, Riaan, S367

Oller, Silvia, S237

Olsson, Anna-Karin, S159, S265

Olsson-Tall, Maivor, S159, S263, S265

Olvet, Doreen, S79

Ongur, Dost, S16

Onitsuka, Toshiaki, S117

Onn, Roy, S339

Ophoff, Roel, S109

Or, Chi Fai, S215

Oranje, Bob, S95, S226, S232, S314

Orens, Sandrine, S420

Oresic, Matej, S68, S69

Oribe, Naoya, S117

Origala, Ajay, S158

Orlov, Natasza, S174, S280

Orlovska, Sonja, S116

Orlovskaya, Diana, S41

Orsini, Nicola, S67, S94

Ortet, Generós, S330

Ortiz, Bruno Bertolucci, S184, S255

Ortiz, Bruno, S96

Ortiz-Gil, Jordi, S145

Ortuño, Felipe, S352

Ortuño, Marta, S352

Ortuño, Noèlia, S423

Os, Jim van, S334

Osborn, Juston, S42

Ose, Claudia, S128

Østefjells, Tiril, S113

Østergaard, Søren D., S342

Ostergaard, Soren Dinesen, S341

Ota, Vanessa, S96, S184

Ouchi, Yasuomi, S277

Overbeek, Greg, S120

Overholser, James, S374

Owe-Larsson, Björn, S120

Owen, Michael J., S273, S379

Owen, Michael, S12, S13, S86, S165

Oyebode, Femi, S280

Ozog, Veronica, S275

Pélaez, Trinidad, S348

Pérez-Solà, Víctor, S237

Pöhö, Päivi, S69

Paccalet, Thomas, S11, S124, S142, S149, S230

Pachas, Gladys, S361

Padberg, Frank, S355

Pagsberg, Anne Katrine, S226, S232, S333

Painter, Michelle, S57

Paker, Benedicto, S244

Paladini, Maria Serena, S202

Paladini, Vittoria, S116

Palaniyappan, Lena, S79, S82, S90, S102, S108, S170, S170, S171, S171, S179, S179, S180, S180, S180, S187, S194, S199, S207, S221, S229, S271, S327, S373, S394

Palao, Diego, S203, S251, S253, S303, S311

Palm, Ulrich, S355

Palmer, Lisa, S247

Palotie, Aarno, S88

Paloyelis, Yannis, S383

Pàmias, Montserrat, S251

Pan Yuen, Hok, S162

Pan, Pedro, S112, S164, S286

Panizzutti, Bruna, S22

Paños, Montse, S253

Pantazopoulos, Charalampos, S235

Pantazopoulos, Harry, S16

Pantelis, Christos, S74, S91, S176, S209, S282, S295

Paola, Presečki, S257

Papaleo, Francesco, S37, S38

Papalino, Marco, S116

Papazova, Irina, S342, S355

Papin, Caroline, S176

Papiol, Sergi, S17, S155

Papp, Mariusz, S202

Papsuev, Oleg, S213

Parak, Mohan, S244

Paramo, Mario, S398

Pardeller, Silvia, S421

Pardeller, Sylvia, S135

Pardo, Marta, S212, S229

Parellada, Mara, S222, S318

Pariante, Carmine M., S118

Pariante, Carmine, S48, S97, S98, S118, S131, S166

Pariente, Jose, S181, S183

Park, Cheol, S411

Park, Jin Young, S188

Park, Joo Eon, S257, S360

Park, Kee Jeong, S232

Park, Kiho, S240

Park, Kyung Tae, S203

Park, Seon-Cheol, S240, S257, S360

Park, Sohee, S29, S46, S47, S77, S195, S196, S421

Park, Sung-Yong, S206, S215, S218, S236, S264, S304, S308, S367, S401

Park, Woon Ju, S29

Parker, Benedicto, S129

Parker, Stephen, S237

Parker, Theresa, S256

Parlar, Melissa, S149

Parnas, Josef, S198, S306

Parola, Alberto, S169, S319

Parrish, Emma, S404

Pasternak, Ofer, S8, S9, S194

Patel, Anita, S87

Patel, Krisna, S92

Patel, Raihaan, S290

Patel, Rashmi, S154, S310

Paterno, Danielle, S10

Patterson, Thomas, S204

Pau, Soldevila-Matias, S384

Paul Amminger, G, S105

Paul Amminger, G., S99, S132, S177, S228

Paulo, Louzada-Junior, S222

Paunio, Tiina, S88

Pauselli, Luca, S167, S178, S201, S259, S409

Pavelková, Lucia, S250

Peña, Javier, S347

Pearlson, Godfrey D., S121

Pearlson, Godfrey, S6, S14

Pedersen, Carsten Bøcker, S59

Pedersen, Carsten, S87, S167

Pedersen, Emilie, S273

Pedrini, Mariana, S2

Pegoraro, Luis Fernando, S146

Pegoraro, Luiz Fernando, S349

Peh, Oon Him, S123, S270, S324

Peitl, Milena, S369

Peitl, Vjekoslav, S369

Pekar, James, S289

Pelayo-Teran, Jose Maria, S373, S374

Pelham, Tanisha, S125

Peña, Javier, S347

Penades, Rafael, S332

Penn, David, S208, S237, S320

Penney, Danielle, S148

Pennuto, Maria, S193

Pennycook, Gordon, S147

Penttilä, Matti, S144

Penzel, Nora, S293

Pepper, Fiona, S13

Peregrino, Antônio, S262

Pereira, Avril, S224

Perez, Jesus, S57

Perez, Maria Isabel Ahuir, S303

Perez, Stephanie, S101

Perez-Iglesias, Rocio, S373

Perfect, Leo, S105

Pergola, Giulio, S100, S116, S193

Perkins, Diana O., S78, S102, S231

Perkins, Diana, S39, S62, S79, S266, S396, S422

Perquier, Florence, S167

Perry, Benjamin, S48

Persson, Mats, S120

Pery, Shani, S65

Peter, Durning, S235

Peters, Benjamin, S250, S387

Peters, Sarah, S220

Petersen, Liselotte, S273

Petersen, Lone, S163

Petra Gronholm, S55

Petros, Natalia, S374

Petrušová, Veronika, S250

Peyre, Hugo, S166

Phahladira, Lebogang, S261, S364, S367

Philip, Mariamma, S181

Philip, Noah, S244

Piccin, Sara, S147

Pichker, Theresia, S135

Pierce, Katherine, S3

Pieters, Lydia, S325

Pignon, Baptiste, S155, S166

Pijnenborg, Marieke (Gerdina), 

Pijnenborg, Marieke, S238, S419

Pikalov, Andrei, S114, S239, S242

Pilar, Javier Gómez, S397

Pilecka, Izabela, S77

Pillinger, Toby, S68, S225, S330

Pilobello, Kanoelani, S235

Pilton, Marie, S321

Pinheiro, Ana, S380

Pinkham, Amy, S208, S320, S358, S360

Pinner, Anita, S298

Pinnock, Farena, S149, S368

Pionke, Renata, S252

Piontkewitz, Yael, S65, S191

Pitt, Michael, S156

Pitta, José Cássio, S375

Plessen, Kerstin J., S336

Plessen, Kerstin, S111, S245, S252, S300

Plessis, Stefan du, S364

Pletnikov, Mikhail, S150

Plewnia, Christian, S355

Plitman, Eric, S290, S329, S393

Pluijm, Marieke van der, S391

Plumley, Hyekyung, S70

Podda, Giovanni, S128

Pogarell, Oliver, S393

Poh, Joann, S281

Polacek, Romana, S65

Polari, Andrea, S378

Poletti, Michele, S46

Pollak, Thomas, S60, S72, S74, S189, S328

Pollock, Bruce, S393

Polo-Velasco, Javier, S237

Pomarol-Clotet, Edith, S145, S174

Pomper, Martin, S8

Poncelet, Patrick, S47

Ponnampalam, Meneshka, S318

Ponsford, Jennie, S260, S359

Porcelli, Annamaria, S85

Porffy, Lilla, S249

Porfy, Lilla, S92

Posselt, Christine Merrild, S365

Pot-Kolder, Roos, S344

Potkin, Steven G., S115, S312

Potvin, Stéphane, S346

Poupon, Cyril, S172

Pour, Benedek, S192

Pousa, ESther, S348

Pousa, Esther, S210, S238, S317, S370, S372, S410

Powell, Susan, S233

Pradhan, Subechhya, S20

Prasad, Konasale, S63

Prats, Claudia, S174

Pratt, Judith, S234

Prayosha Villa, L., S305

Preisig, Martin, S254

Prener, Christopher, S137

Preskorn, Sheldon, S63

Price, Jack, S105

Pries, Lotta-Katrin, S84, S160

Prikken, Merel, S77, S95

Prinzio, Patricia Di, S365

Pritchard, Megan, S72

Pross, Benjamin, S393

Provenzani, Umberto, S383

Psicotici, Gruppo Regionale Esordi, S426

Puchalski, Severin, S363

Puhl, Matthew, S2

Puig-Navarro, Olga, S123, S229

Pujol, Nuria, S237

Pulasky, Beth, S248

Pulkkinen, Ulla, S349

Pungor, Katalin, S130, S311

Punzi, Giovanna, S85

Purdon, Scot, S295

Purr, Franziska, S136

Purves-Tyson, Tertia, S64, S64

Qi, Zhenghan, S104

Qian, Yun, S333

Qian, Zhenying, S223

Qiyong, Gong, S331

Quarto, Tiziana, S116

Quattrocchi, Graziella, S416

Quattrone, Diego, S7, S59, S75, S110, S158, S246, S258, S354

Qubad, Mishal, S387

Quide, Yann, S347

Quigley, Harriet, S59

Quinn, Anne Marie, S130

Quintana, Daniel, S362

Räikkönen, Katri, S26

Rösler, Lara, S250, S387

Rössler, Wulf, S376

Rødevand, Linn, S205, S362

Røssberg, Jan Ivar, S113

Raballo, Andrea, S46

Racette, Elise, S362

Rachael, Spooner, S105

Rachel, Jenkins, S235

Radaideh, Ali, S180, S180

Radhakrishnan, Rajiv, S160

Radin, Duje, S29

Radin-Gilboa, Shulamit, S54

Radnovich, Alexander, S63

Radua, Joaquim, S221, S327

Rae, Charlotte, S173

Raffard, Stéphane, S359

Ragazzi, Taciana, S166

Ragland, J. Daniel, S4

Rahel, Flückiger, S407

Raij, Tuukka, S80, S172

Raitakari, Olli, S272

Rajarajan, Prashanth, S82

Rakhmanova, Valentina, S41

Ramaekers, Jan, S182

Ramella-Cravaro, Valentina, S383

Ramikie, Teniel, S235

Rampino, Antonio, S100, S116, S193

Ramsay, Hugh, S272

Ramsey, Amy, S19

Rand, David, S147

Randers, Lasse, S95

Ranganathan, Mohini, S27

Rannikko, Irina, S144

Rao, Naren, S181, S309

Rapisarda, Attilio, S123, S270, S324

Rapoport, Judith, S34

Rapp, Alexander, S136

Rask, Charlotte Ulrikka, S103, S334

Ratheesh, Aswin, S105, S162, S270, S376

Rathi, Yogesh, S194

Ratner, Yael, S198

Rau, Carina, S95

Rauchmann, Boris, S17

Rauschenberg, Christian, S160, S219

Rautio, Nina, S144

Ray, Jim, S339

Razzino, Eugenio, S204, S312, S414

Reay, William, S296

Recasens, Marc, S279, S282, S385

Recio, Sandra, S226

Reckziegel, Ramiro, S288

Redondo, Marina, S125, S181, S229

Reese, Elaine, S335

Reeves, Gloria, S43

Refisch, Alexander, S152

Refsum, Helge, S122

Rehen, Stevens K., S15

Reichard, Holly, S339

Reichenbach, Juergen, S121, S221

Reichenberg, Abraham, S39, S77

Reichenberg, Avi, S40, S98, S165, S247, S396

Reid, Meredith, S41, S120

Reid, Vincent, S301

Reif, Andreas, S30, S250, S387

Reilly, Thomas, S56

Reinares, Maria, S5

Reinhart, Robert, S303

Reininghaus, Ulrich, S6, S6, S7, S158, S160, S219, S307

Reixach, Elisenda, S208

Remington, Gary, S104, S115, S267, S290, S306, S309, S309, S313, S329, S354, S393, S413, S415

Rempfer, Melisa, S362

Remus, Isadora, S288

René,Müri, S184

Reneman, Liesbeth, S391

Reniers, Renate, S98, S209, S280, S328, S334, S370

Reponen, Elina J., S205

Reponen, Elina Johanna, S362

Reser, Maree, S303, S406

Ressler, Kerry, S235

Revu, Omkar, S150

Reymond, Annina R., S239

Reynolds, Gavin, S329

Reznik, Noa, S306

Rhebergen, Didi, S157

Ribeiro, Wagner, S55

Rice, Simon, S4

Richard, Jean-Romain, S155

Richards, Alex, S7

Richetto, Juliet, S65, S127

Riecher-Rössler, Anita, S99, S116, S132, S199, S228, S378

Riehle, Marcel, S410

Rietschel, Marcella, S343

Rigoux, Lionel, S314

Rikandi, Eva, S80, S172, S285

Rimvall, Martin K., S103, S334

Ripka, Stephan, S287

Ripoll, Oleguer Plana, S87

Ris, Laurence, S20

Ritsner, Michael S., S198

Riva, Marco A., S297

Riva, Marco Andrea, S202

Rivolta, Davide, S340

Rizvi, Asra, S100, S267

Rizzo, Gaia, S83

Roach, Brian, S266

Roalf, David, S57

Robbins, Trevor W, S360

Roberto Bakker, P., S157

Roberts, Ruth, S118

Roberts, Susanna, S25

Robertson, James, S194

Robertson, Sharman, S216

Robinson, Delbert, S330

Robinson, Joshua F., S85

Robson, Philip, S27

Robson, Siân, S171

Roché, Matthew, S401

Rocha, Deyvis, S161, S184, S255, S375

Roche, Ayesha, S370

Roder, Volker, S239

Rodrigues, Naielly, S21

Rodriguez, Carolyn, S2

Rodriguez, Katrina, S190

Rodriguez, Vicente Molina, S205, S249, S397

Rodriguez, Victoria, S59, S246, S354

Rodriguez-Raecke, Rea, S277

Roeh, Astrid, S342

Roepstorff, Andreas, S350

Rogdaki, Maria, S81, S291, S395

Rohde, Luis Augusto, S286

Roine, Timo, S279, S395

Roiser, Jonathan, S43

Rokita, Karolina, S325

Rolls, Asya, S47

Romain, Ahmed Jérôme, S17

Romano, Raffaella, S100, S116

Romanowska, Sylvia, S355

Romero Jiménez, Rodrigo, S259

Romm, Kristin L., S113

Roos, Johannes, S399

Rosa, Ayesa-Arriola, S184

Rosa, Eduarda, S22

Rosa,Ayesa Arriola, S259

Rosana, Shuhama, S166, S222

Rosell, Daniel, S240

Rosen, Marlene, S114, S144, S147, S209

Rosen, Raymond, S139

Rosenbrock, Holger, S380

Rosengard, Rachel, S56, S185

Rosenheck, Robert, S320

Rosenthal, Adi, S4

Ross, Christopher, S289

Rossell, Susan, S78, S137, S138, S139, S143, S246, S252, S260, S279, S302, S303, S321, S355, S359, S406

Rossetti, Andrea Carlo, S202

Rossi, Menezes Paulo, S222, S227

Rossler, Wulf, S195

Rossolatos, David, S295

Rostamzadeh, Ayda, S285

Rostrup, Egill, S80, S83, S119, S224, S289, S383, S387

Rot, Marije aan het, S238, S260

Rothbaum, Maya, S198

Rothmond, Debora, S64

Rothshield, Lily, S198

Rothwell, John, S280

Roussos, Panos, S188

Rovó, Zita, S395

Rovira, Esther Lorente, S317

Rowe, Monique, S218

Rowland, Emma, S374

Rowland, Laura, S18, S20, S186

Rowland, Toby, S48

Roy, Martin, S254

Rubia, Katya, S174

Rubinstein, Katya, S165

Rubio, Jose, S93, S207

Ruderfer, Douglas, S34

Ruef, Anne, S114, S119, S155, S168, S185, S246, S292, S316

Ruggero, Camilo, S40

Ruhrmann, Stephan, S209, S285, S328

Ruiz, Ada, S370, S410

Ruiz, Isabel, S238, S348, S370, S372

Ruiz-Delgado, Isabel, S317

Rujescu, Dan, S51, S85

Rund, Bjørn Rishovd, S253

Rund, Bjorn, S245, S401

Ruparel, Kosha, S57

Ruppa, Markku, S345

Rusjan, Pablo, S390

Russell, Anthony, S178

Russell, David, S176

Russo, Debra A, S57

Russo, Manuela, S98

Russon, Penni, S4

Ruth, Zhou De-Hui, S427

Rutigliano, Grazia, S56, S162, S383

Rutten, Bart P. F., S166

Rutten, Bart, S53, S72, S82, S84

Ruth, S259

Richetto, S49

Ryan, Alex, S61

Rydkjaer, Jacob, S226, S232

Sönmez, Nasrettin, S113

Søndergaard, Anne, S245, S300, S336

Saarela, Maria, S68

Saarinen, Maiju, S272

Sabesan, Priyadharshini, S221, S327

Sabhwerwal, Sophie, S53, S72

Sadeh, Elham, S216

Saha, Sukanta, S237

Sahakian, Barbara J., S275

Saia-Cereda, Veronica M., S15

Sailas, Eila, S32

Saillard, Jelena, S236, S341

Saini, Suriati, S295

Saiz-Martinez, Pilar, S119, S222, S420

Sakakibara, Eisuke, S381

Sakuma, Kei, S315

Sakurada, Hanako, S381

Salas, Miriam, S238

Salcedo, Tatiana Oviedo, S261

Salgado, Joao, S22

Salgado-Pineda, Pilar, S174

Salibi, Noah, S120

Salisbury, Dean, S288, S390

Salokangas, Raimo K.R., S272

Salokangas, Raimo, S144, S147, S209, S328, S391

Saltó, Carme, S113

Salum, Giovanni, S164

Salvador, Teresa, S237

Salyers, Michelle, S343

Sambataro, Fabio, S83

Sami, Musa, S259

Samuel, Sarrazin, S172

SanDiego, Christine, S176

Sanchez Blanco, Lucía , S259

Sanchez, Stella, S286

Sanchez-Cabezudo, Dolores, S410

Sanchez-Gistau, Vanessa, S303

Sanchez-Gutierrez, Teresa, S255

Sanchez-gistau, vanessa, S253

Sand, Michael, S139, S248, S353, S425

Sanders, Caroline, S106

Sandin, Sven, S77

Sandra, Iglesias, S72

Sandvik, Leiv, S113

Sanfelici, Rachele, S185, S246

Sanja,Devčić, S257

Sanjuan, Julio, S157, S200, S276, S424

Santesteban-Echarri, Olga, S151, S231

Sanz-Fuentenebro, Francisco Javier, S205

Saperia, Sarah, S306

Saponaro, Alessio, S426

Sarkar, Abhirup, S328

Sarkar, Nilanjan, S77, S195, S196, S421

Sarkkinen, Katja, S345

Sarno, Elaine Di, S262

Sarnyai, Zoltan, S326

Sarró, Salvador, S145

Sarris, Jerome, S405

Sartori, Giuseppe, S393

Sartorio, Crocettarachele, S354

Sartory, Gudrun, S128

Sasabayashi, Daiki, S394

Saskia,Jünger, S285

Sass, Louis, S46

Satlin, Andrew, S236, S341

Sato, Hideyo, S20

Sato, João Ricardo, S184

Satomura, Yoshihiro, S381

Satterthwaite, Theodore, S57

Sauer, Andreas, S340

Sauer, Heinrich, S287

Sauve, Genevieve, S148

Savadijev, Peter, S184

Savadjiev, Peter, S194

Savage, Christina, S264, S353

Savill, Mark, S4

Savulich, George, S351

Sawa, Akira, S8

Sawada, Ken, S290

Sbrini, Giulia, S202

Scarborough, Joseph, S127

Scardoelli, Maria, S262

Scarpazza, Cristina, S92, S331, S393

Scarr, Elizabeth, S70

Schäfer, Miriam R., S228

Schäfer, Miriam, S132

Schäppi, Lea, S194, S198

Schæbel, Helle, S424

Schöbi, Dario, S72, S281, S331

Schack, Stephan, S221

Schaefer, Miriam, S99, S116, S326

Schafer, Miriam, S378

Schalbroeck, Rik, S104

Schalit, Liat, S306

Schaum, Michael, S250, S387

Scheepers, Floor, S92

Scheffler, Freda, S261, S367

Scheffler, Frederika, S364

Scheller, Bertram, S340

Schieveld, Jan, S219

Schifani, Christin, S43

Schiffman, Jason, S43

Schirmbeck, Frederike, S107, S269

Schlögelhofer, Monika, S99, S132, S228

Schlagenhauf, Florian, S172, S278

Schlogelhofer, Monika, S116, S378

Schmidt, André, S102, S199, S281

Schmidt, Andre, S383

Schmidt, Lyndsay, S305

Schmidt, Stefanie, S228

Schmitt, Andrea, S17

Schnack, Hugo, S101, S287

Schneider, Karoline, S284

Schneider-Axmann, Thomas, S343

Schneider-Thoma, Johannes, S115, S418

Schooler, Nina, S216, S320

Schoretsanitis, Georgios, S198

Schork, Andrew, S85

Schröder, Johannes, S115, S140, S289

Schreuder, Marieke, S14

Schruers, Koen, S76

Schulte, Raphael, S67, S239

Schultze-Lutter, Frauke, S109, S209, S228, S407

Schulz, Steffen, S152

Schulze, Thomas G., S155

Schumann, Andy, S152

Schurhoff, Franck, S155, S160

Schutte, Maya, S109, S173

Schwan, Raymund, S169

Schwannauer, Matthias, S279, S282, S340, S409

Schwannauer, Matthis, S385

Schwartenbeck, Philipp, S385

Schwartze, Michael, S380

Schwenk, Jochen, S120

Schwippel, Tobias, S355

Schwitzer, Thomas, S169

Scodes, Jennifer, S107

Scott, James, S61, S237, S379

Seabra, Gabriela, S297

Seccomandi, Benedetta, S196

Secher, Rikke Gry, S163

See, Yuen Mei, S309

Seedat, Soraya, S364

Segan, Catherine, S151

Seibyl, John, S176

Seidel, Jan, S85

Seidl, Ulrich Seidl, S140

Seidl, Ulrich, S289

Seidman, Larry J., S78, S79, S90, S117, S194, S223, S231, S235, S366

Seidman, Larry, S39, S54, S62, S102, S104, S236, S266, S396, S422

Seifritz, Erich, S81, S174, S284

Selten, Iris, S226

Selten, Jean-Paul, S67, S104, S256, S341

Selvaggi, Pierluigi, S83

Seminerio, Fabio, S258, S354

Sendt, Kyra-Verena, S382

Seo, Ho Jun, S307

Seo, Ho-Jun, S242

Seo, Hye-Jin, S201

Seppälä, Annika, S144

Seppälä, Jussi, S144, S168

Serpa, Mauricio Henriques, S376

Serpa, Mauricio, S133, S195

Servaas, Michelle, S177

Seth, Anil, S173

Setién-Suero, Esther, S251, S268

Setiaman, Nikita, S287

Setien-Suero, Esther, S92, S142, S184, S373, S374, S392

Seves, Mauro, S114, S209, S285

Sevigny, Jeffrey, S340

Sevilla-Llewellyn-Jones, Julia, S151

Sevy, Serge, S244

Shafer, Anna, S288

Shafner, Laura, S134

Shah, Jai, S56, S182, S185

Shah, Parita, S329

Shalhoub, Huda, S241

Sham, Pak, S7, S158, S354

Shan, LeeAnn, S264, S353

Shang, Jing, S283

Shannon, Ciaran, S317

Shapiro, Dan, S54

Shapiro, Daniel I., S36, S78, S366

Sharma, Shivani, S220

Shatzi, Vasiliki, S50

Shen, Dinggang, S103

Shenton, Martha E., S36, S104, S366

Shenton, Martha, S90, S117

Shergill, Sukhi, S92, S164, S174, S249, S280, S383

Shetty, Hitesh, S411

Shi, Hai-song, S244

Shi, Li-juan, S333

Shi, Yan, S331

Shimada, Takamitsu, S248

Shin, Il-Seon, S411

Shinde, Mayura, S425

Shivakumar, Venkataram, S345

Shon, Seung-Hyun, S219, S387, S391

Shon, Seung-hyun, S287

Short, Sarah, S103

Shuhama, Rosana, S227

Shukla, Ayushi, S181

Shum, Carol, S105

Shungu, Dikoma, S2

Siamouli, Melina, S393

Siddiqui, Ishraq, S306

Sideli, Lucia, S75, S258, S354

Sidovar, Matt, S425

Siemerkus, Jakob, S331

Siettou, Eleni, S243, S348

Sigvard, Anne, S224, S383, S387

Silva, Nicole, S21

Silva, Tania Da, S390

Silvana, Jelavić, S257

Silverman, Bernard, S312, S413

Silverstein, Steven, S10, S139, S140, S401

Sim, Kang, S123, S324

Sim, Xueling, S12

Simmons, Adam, S312, S413

Simons, Claudia, S269

Simonsen, Arndis, S169, S350

Simonsen, Erik, S272

Sing, Wong Siu, S427

Singer, Wolf, S340

Singh, Arun, S130, S311

Sinkeviciute, Igne, S95, S261

Sintes, Anna, S229

Siskind, Dan, S66, S178

Siu, Cynthia, S242, S267

Sivapalan, Sudhakar, S295

Sjöstedt, Evelina, S120

Sjöström, Nils, S211, S212, S305, S407

Skidmore, Frank, S91

Sklar, Pamela, S34

Skosnik, Patrick, S27

Skovgaard, Anne Mette, S103, S334

Skuban, Aleksandar, S94

Skurnik, Norbert, S167

Skymba, Haley, S4

Slavu, Ruxandra, S160

Slezak, Diego Fernández, S43

Slifstein, Mark, S2, S71

Slikboer, Reneta, S406

Sloan, Richard, S17

Smallman, Richard, S131, S148

Smelror, Runar, S306

Smesny, Stefan, S116, S121, S122, S132, S221, S228, S378

Smesny, Stephan, S99, S228

Smith, Adam, S139

Smith, Eleanor, S301

Smith, Geoffrey, S291

Smith, George Davey, S165, S273, S379

Smith, Shubulade, S87

Smith, Thomas, S107, S108

Snodgress, Matthew, S47, S77

So, Joyce, S296, S399

So, Suzanne, S161

Soares, Bernardo, S130

Soares, Walter, S378

Solberg, Dag, S122

Soldevila-Matias, Pau, S200, S276

Soldevilla, Pablo, S157

Sole, Montserrat, S253, S303

Solida, Alessandra, S389

Somes, Nathaniel, S90, S194

Sommer, Iris E, S95

Sommer, Iris, S51, S67, S109, S173

Song, In Chan, S186

Song, Min-Kyu, S161, S218, S236, S264, S296, S304, S308, S326, S367

Sonnenschein, Susan, S338

Soo, Kwon Jun, S330

Sood, Poonam, S87, S166, S246

Sorg, Christian, S283

Sormunen, Elina, S272

Sotelo, Eva, S249, S392

Soto, Marta, S417

Spang, Katrine Søborg, S245, S336

Spang, Katrine S., S300

Spang, Katrine, S110, S252

Spaniel, Filip, S173

Sparano, Olga, S157, S200, S276

Spencer, Kevin M., S117

Spinz, Rodrigo, S288

Spiros, Athan, S294, S411

Spoorthy, Mamidipalli, S322, S405

Sportel, Bouwina, S260

Srihari, Vinod, S272, S426

Sriretnakumar, Venuja, S296, S399

Srivastava, Deepak, S105

Staal, Nina, S333

Stabell, Lena, S346

Stadsgaard, Henriette Brockdorff, S336

Stadsgaard, Henriette, S252

Stafford, Jean, S377

Stahl, Daniel, S56, S87

Stain, Helen, S321

Stainton, Alexandra, S357

Stansley, Branden, S70, S127

Starace, Fabrizio, S426

Stavely, Heather, S321

Steen, Nils E., S205

Steen, Nils Eiel, S227, S362

Steen, Vidar M., S227

Steen, Vidar Martin, S358

Stefan, Just, S143

Stefan, Kaiser, S174

Stefanics, Gabor, S281

Steffens, Maria, S119, S386

Stegmayer, Katharina, S175, S184, S194, S198

Steichen, Conny, S239

Steiner, Johann, S40

Stenstrøm, Anne Dorte, S333

Stephan, Klaas Enno, S72, S254, S278, S281, S314, S331

Stepien, Marta, S284

Steullet, Pascal, S1, S395

Stewart, Robert, S72, S84, S154, S411

Stewart, Sherry, S153

Stilo, Simona, S166, S246

Stip, Emmanuel, S17

Stochl, Jan, S48, S57, S379

Stockmeier, Craig, S374

Stockton-Powdrell, Charlotte, S106

Stoeter, Peter, S277

Stojanovski, Sonja, S226

Stolz, Erin, S2

Stone, James, S81, S328

Stone, William S., S36, S78, S90, S102, S104, S223, S366

Stowkowy, Jacqueline, S231

Strahimir, Sučić, S257

Straub, Richard, S85

Straube, Benjamin, S195

Strik, Werner, S184

Stringer, Dominic, S87

Strippoli, Marie-Pierre, S254

Strube, Wolfgang, S343, S355, S393, S406, S416

Stubberud, Jan, S356

Stubbs, Brendon, S87, S330, S405

Sturm, Elisabeth, S388

Styner, Martin, S194

Su, Wayne, S291

Subotnik, Kenneth L., S136

Subotnik, Kenneth, S18

Subramaniam, Karuna, S278

Subramanian, Siva, S235

Suckling, John, S131, S148

Suen, Yi Nam, S138, S214, S215, S372

Sugar, Catherine, S216, S320

Sugranyes, Gisela, S123, S181, S183, S229, S337

Sullivan, Courtney, S19, S19

Sultana, Tania, S129

Sumiyoshi, Chika, S419

Sumiyoshi, Tomiki, S419

Sumner, Philip, S78, S137, S143, S246, S279

Sun, Bin, S192

Sun, Xiaoqi, S161

Sundar A, Shyam, S136

Sundram, Suresh, S224

Sundvall, Jouko, S285

Suokas, Kimmo, S88

Surcel, Heljä-Marja, S272

Surti, Toral, S142

Susser, Ezra, S271

Sutterland, Arjen, S86

Suvisaari, Jaana, S68, S69, S80, S88, S172, S285, S349

Suvitaival, Tommi, S69

Suzuki, Hirobumi, S412

Suzuki, Kazunori, S412

Suzuki, Michio, S394

Suzy, Harrigan, S162

Svendsen, Ingrid, S410

Svenning, Jens-Christian, S59

Swami, Viren, S368

Sweeney, John A., S121

Sweeney, John, S6, S14

Sweeney, Rohan, S151

Sweeney, Shaun, S408

Swillen, Ann, S86

Szöke, Andrei, S166

Szele, Francis, S192

Szoke, Andrei, S155, S160, S167

Tak, Erwin, S407

Taka-Eilola, Tiina, S164

Takahashi, Tsutomu, S394

Takao, Hensch, S1

Takayanagi, Yoichiro, S394

Takegoshi, Yusuke, S394

Takei, Nori, S277

Takeuchi, Hiroyoshi, S267, S309, S354, S413

Talkowski, Michael, S33

Talor, Monica, S190

Tamagnan, Gilles, S176

Tamburri, Paul, S340

Tamminga, Carol A., S121

Tamminga, Carol, S6, S14

Tan, Eric, S78, S137, S139, S143, S246, S252, S321, S359

Tan, Wilson, S130

Tanase, Costin, S126

Tandi, Jesisca, S281

Tandon, Rajiv, S241

Tang, Charmaine, S309

Tang, Yee Man, S372

Tang, YinYin, S90

Tang, YingYing, S36, S366

Tang, Yingying, S104, S223

Tang, Yun-Xiang, S121

Tangmose, Karen, S224, S383, S387

Tanguy, Audrey, S268, S420

Tania,Kümpfel, S261

Tanja,Franćeski, S369

Tanskanen, Antti, S379

Tao, Haojuan, S187

Tardif, Christine, S182

Tarquis, Corentin, S420

Tarricone, Ilaria, S75, S123

Taylor, Adam, S27

Taylor, David, S310

Taylor, Jennifer, S61

Taylor, Matthew, S310

Taylor, Valerie, S413

Teasdale, Scott, S405

Teigset, Charlotte, S245, S401

ten Have, Margreet, S160

ten Kate, Corine, S341

Tenan, Silvia, S166

Tenback, Diederik, S407

Teng, Irving, S214

Teo, Celine, S104

Termorshuizen, Fabian, S104

Teufel, Christoph, S377

Thanju, Amod, S275, S408

Theberge, Jean, S179, S179, S221, S327

Theo, Broekman, S239

Theochari, Eirini, S243, S348

Therman, Sebastian, S349

Thermenos, Heidi, S194

Therrien, Francois, S133

Thien, Kristen, S162, S270, S376

Thiery, Evert, S84

Thimm, Jens, S324

Thirthalli, Jagadisha, S136

Thomann, Philipp, S140, S289

Thomas, Anthony, S91

Thomas, Elizabeth, S137, S143, S246

Thomas, McGlashan, S422

Thomas, Megan, S301

Thomas, Neil, S78, S260, S302

Thompson, Andrew, S99, S116, S132, S228, S321, S378

Thompson, Judy, S10

Thompson, Wesley, S85

Thomson, David, S234

Thomson, Sumana, S137

Thorndike, Anne, S361

Thornton, Allen, S291

Thorup, Anne A.E., S336

Thorup, Anne Amalie, S111, S245, S300

Thorup, Anne, S111, S163, S252

Thuné, Hanna, S279, S282, S385

Tian, Ye, S282, S295

Tibbo, Philip, S153, S295

Tiemeier, Henning, S231, S402

Tiihonen, Jari, S67, S88

Tikkala, Jenni, S345

Timmerman, Marieke, S238

Tingue, Samantha, S121

Tiwari, Arun, S115, S218, S412

Tobler, Philippe N., S81, S284

Tocco, Michael, S114, S239, S242, S353

Tognin, Stefania, S393

Toh, Wei Lin, S78, S252

Töhönen, Tuomo, S345

Tomasetti, Carmine, S313

Tomiello, Sara, S72, S281, S314, S331

Ton, Trang, S420

Tonsig, Daniela Koga, S375

Tor, Jordina, S183, S229

Tordesillas-Gutiérrez, Diana, S184, S259, S392

Tordesillas-Gutierrez, Diana, S92, S142, S251, S268

Torgalsboen, Anne-Kari, S141, S250, S253

Torniainen-Holm, Minna, S88, S285, S349

Torregrossa, Lénie, S47, S77, S195, S196, S421

Torrent, Carla, S222

Torretta, Silvia, S100, S193

Torrey, E. Fuller, S24

Tortelli, Andrea, S167

Tortorella, Alfonso, S201, S363

Tost, Meritxell, S251

Toutountzidis, Diamantis, S220

Touya, Maelys, S97

Towell, Tony, S368

Trabelsi, Rouaa, S163, S423

Traber-Walker, Nina, S230, S336

Tracy, Derek, S280

Tran, Nga, S214

Tréhout, Maxime, S396

Tripoli, Giada, S7, S59, S75, S110, S158, S246, S258, S354

Tröger, Laura, S168

Trotta, Antonella, S59, S97, S166, S246

Trudeau, Jeremiah, S139

Tsai, Joyce, S242

Tsaltas, Eleftheria, S243, S348

Tse, Desmond, S182

Tseng, Huai-Hsuan, S389

Tseng, Wen-Yih Isaac, S186

Tsirogiannis, Constantinos, S59

Tsuang, Ming T., S78, S231

Tsuang, Ming, S39, S62, S79, S102, S266, S396, S422

Tsujino, Naohisa, S315

Tucha, Oliver, S177

Tully, Laura, S3, S3

Tunca, Zeliha, S356

Tuominen, Lauri, S391

Turetsky, Bruce, S57

Turkheimer, Federico, S81, S83

Turner, Alyna, S151

Turner, Luana, S18

Tuulio-Henriksson, Annamari, S88

Tuval-Maschiach, Rivka, S165

Tyler, Teadora, S192

Tzivoni, Yair, S306

Tzoumakis, Stacy, S229

Uchino, Takashi, S315

Uehara, Takashi, S248

Ueland, Torill, S356

Ugolini, Annarosa, S412

Uher, Rudolf, S252

Uhlén, Mathias, S120

Uhlhaas, Peter, S2, S279, S282, S340, S385, S409

Ulas, Halis, S368

Ullrich, Johannes, S254

Ulshen, Danny, S159

Umbricht, Daniel, S340

Umeh, Annabel, S171

Ümit,Tural, S316

Undurraga, Juan, S202, S269, S364

Unruh, Leonhardt, S327

Unsöld, Svenja, S128

Upthegrove, Rachel, S48, S48, S131, S144, S147, S148, S159, S209, S209, S280, S283, S328, S357, S370

Uranova, Natalya, S41

Urban, Alexander, S33

Uriarte, Jose Juan Uriarte, S205

Urquijo, Maria F., S119, S386

Urquijo, Maria Fernanda, S144

Ursini, Gianluca, S82, S85

Usall, Judith, S113, S208, S212, S370, S410

V. J. Ramesh,, S136

Vahdani, Bita, S344

Väisänen, Heli, S345

Vakhrusheva, Julia, S17

Vakulin, Andrew, S284

Valiengo, Leandro, S133

Valmaggia, Lucia, S307

Van Der Water, Judy, S126

Van Haren, Neeltje, S77, S109, S242

Van Harten, Peter N., S325

Van Harten, Peter, S295

Van Keuren-Jensen, Kendall, S221

Van Os, Jim, S295

Van Rheenen, Tamsyn, S176

Van Snellenberg, Jared, S44

Van de Bilt, Martinus, S133

Van der Gaag, Mark, S344

Van der Zalm, Yvonne, S67

Vanbellingen, Tim, S184

Vandeleur, Caroline, S254

Vandenberghe, Frederik, S310

Vandevelde, Anaïs, S396

Vanes, Lucy, S92, S383

Vanover, Kimberly, S236, S320, S341

Vaquero, Mercedes, S249, S392

Varambally, Shivarama, S181

Varela, Eva, S123

Vargas, Teresa, S42

Varley, James, S61

Vasconcelos, Selene, S262

Vasile, Daniel, S27

Vaskinn, Anja, S253

Vassilouli, Spyridoula, S243, S348

Vassos, Evangelos, S7, S59, S158, S246

Vaz, Stephanie McDermid, S149

Vázquez-Bourgon, Javier, S184, S259

Veddum, Lotte, S336

Vedul-Kjelsås, Einar, S113

Veijola, Juha, S88, S101, S164, S272

Vekich, John, S412

Velarde, Amalia, S72

Velden Hegelstad, Wenche ten, S153

Veling, Wim, S271, S344, S419

Velligan, Dawn, S30, S31, S404

Vellucci, Licia, S313

Velthorst, Eva, S39, S39, S40, S266, S269

Venkatasubramanian, Ganesan, S181, S345

Ventura, Joseph, S18, S136

Verbruggen, Lise, S20

Verde, Pablo, S343

Verdu, Elena, S190

Veres, Daniel, S192

Vergaelen, Elfi, S86

Verhulst, Frank, S103, S334

Verma, Swapna, S99, S116, S132, S228, S378

Vermeulen, Jentien, S86

Vernal, Ditte Lammers, S333

Vernon, Anthony, S65, S191

Veronese, Mattia, S81, S83, S291, S395

Vestergaard, Mogens, S88

Vicente, Molina, S392

Vidrih, Branka, S369

Viechtbauer, Wolfgang, S341

Vieira, Sandra, S92, S374

Viertio, Satu, S32

Vieta, Eduard, S5, S318

Vignapiano, Ananrita, S264

Vignapiano, Annarita, S263

Viher, Petra, S184, S194

Viikari, Jorma, S272

Vikhreva, Olga, S41

Vila-Badia, Regina, S113, S210, S212

Vilaplana, Claudia, S251

Vilella, Elisabet, S253, S303

Villagonzalo, Kristi-Ann, S302

Villalobos, Anna, S176

Villaplana, Anna, S210

Villarreal, Mirta F., S286

Villers, Agnès, S20

Vilme, Mike, S361

Vilner, Yael, S339

Vincent, Andrew, S284

Vincent, Angela, S141

Vincent, John, S296

Vingerhoets, Claudia, S182, S275, S391

Vink, Matthijs, S225

Vinkers, Christiaan H., S109

Vinkers, Christiaan, S82

Vinogradov, Sophia, S278

Virdee, Gursharan, S31

Viviano, Joseph, S176, S226

Vizzotto, Adriana, S262

Vohs, Jennifer, S319

Voineskos, Aristotle, S115, S176, S226, S306, S422

Volkow, Nora, S45

Vollenweider, Franz X, S281

Vorstman, Jacob, S247, S376

Voss, Andreas, S152

Voutilainen, Greta, S349

Vreira, Sandra, S331

Vucurovic, Ksenija, S381

van Amelsvoort, Therese, S86, S183, S275

van Borkulo, Claudia, S96

van Dijk, Floor, S107

van Donkersgoed, Rozanne, S260

van Dorsselaer, Saskia, S160

van Duin, Esther, S182

van Eindhjoven, Phillip, S183

van Elst, Ludger Tebartz, S11

van Erp, Theo, S396

van Haren, Neeltje, S225, S287

van Harten, Peter, S407

van Opstal, Daniel P.J., S101

van Os, Jim, S7, S76, S84, S103, S117, S158, S160, S166, S219

van Oudenaren, Mathilde, S182

van Praag, Cassandra Gould, S173

van Rooijen, Geeske, S86

van Themaat, Anna Hester Ver Loren, S300

van Tol, Marie-José, S177

van de Bilt, Martinus Theodorus, S195, S376

van de Chapelle, Chuck, S233

van de Kerkhof, Marita, S86

van den Bree, Marianne, S86

van der Burg, Nadine, S295

van der EL, Kristi, S321

van der Gaag, Mark, S238

van der Leeuw, Christine, S117

van der Ley, Claude, S117

van der Merwe, Nicolaas, S399

van der Weiden, Anouk, S77

van-der-EL, Kristi, S404

von Nielander, Luisa, S112

von Plessen, Kerstin, S111, S300

Wade, Joshua, S77, S195, S196, S421

Waern, Marda, S305

Waern, Margda, S211, S212, S407, S426

Wagner, Michael, S386

Wainsztein, Agustina, S286

Waite, Felicity, S335

Wakuda, Tomoyasu, S277

Walitza, Susanne, S230, S336

Walker, Elaine F., S78, S102, S231

Walker, Elaine, S35, S39, S62, S79, S266, S396, S422

Wall, Matthew, S385

Wall, Melanie, S107

Wallace, Anna, S425

Walling, David, S139, S340

Walters, James, S165, S273, S379

Walther, Mario, S121

Walther, Sebastian, S42, S175, S184, S194, S198

Waltz, James, S43

Wan, Claire Ramsey, S259

Wang, Changming, S329

Wang, Chenhao, S281

Wang, Chuan-Yue, S352

Wang, Chuanyue, S241, S329

Wang, Hee Ryung, S201

Wang, JiJun, S90, S223, S366

Wang, JianLi, S355

Wang, Jijun, S36, S104

Wang, Junjie, S223, S366

Wang, Qiang, S90, S394

Wang, Yabing, S295

Wang, Yen-Chin, S274

Wang, Yi, S244

Wang, Yiming, S390

Wang, Yingcheng, S90, S394

Wang, Yunpeng, S85

Wannan, Cassandra, S176

Ward, Philip, S405

Wartelsteiner, Fabienne, S421

Waterreus, Anna, S365

Waters, Patrick, S61

Watkins, Melanie, S343

Watmuff, Bradley, S400

Watson, Andrew, S148

Webb, Roger T., S167

Webb, Roger, S388

Weber, Lilian, S72, S281, S331

Weber-Stadlbauer, Ulrike, S65, S234

Webster, Maree, S38

Wegelius, Asko, S88, S345

Wei, Deng, S90, S394

Weibell, Melissa, S153

Weickert, Cynthia Shannon, S24, S38, S64, S73, S74

Weickert, Thomas, S73

Weijers, Jonas, S341

Weimand, Bente, S211

Weinberger, Daniel R., S82

Weinberger, Daniel, S85

Weiner, Ina, S65, S191

Weingart, Michal, S236, S341

Weis, Laura, S368

Weiser, Mark, S23, S23, S165

Weiske, Johanna, S168

Weiss, Catherine, S133, S241

Weizman, Abraham, S339

Welikson, Tamara, S136

Wells, Ruth, S73

Wellstein, Katharina, S254, S314

Wen, Zhexing, S299

Wenneberg, Christina, S119

Werbeloff, Nomi, S23

Werge, Thomas, S85

Werner, Lisa, S11

Westerhausen, Rene, S293

Wexler, Bruce, S142

Wexler, Deborah, S361

Wheeler, Anne, S226

Whelan, Pauline, S106

White, Amanda, S37

White, David, S41, S91, S120

Whitfield-Gabrieli, Susan, S90, S104, S194

Whittingham-Dowd, Jayde, S233, S234

Wibral, Michael, S250, S340, S387

Wichers, Marieke, S14, S84, S124

Widman, Linnea, S67

Wiest, Roland, S175, S184

Wiffen, Ben, S246

Wigman, Hanneke, S14

Wigman, Johanna, S96, S334

Wigton, Rebekah, S249

Wijtenburg, Andrea, S20, S186

Wilkin, Jennifer, S104

Willi, Cosima, S233

William, Lo Tak-Lam, S427

Williams, Jill, S151

Williams, Lana, S411

Williams, Leanne, S192

Williams, Stephen, S131, S148

Williams, Steve, S118, S413

Williams, Steven CR, S83, S89

Wilson, Alan, S43, S390

Wilson, Clayton, S149

Wilson, Nathan, S78

Wilson, Robert, S61

Wilson, Robin, S28, S154, S384

Wiltfang, Jens, S128

Winckel, Karl, S178

Wingård, Louise, S120

Winje, Dagfinn, S324

Winkel, Ruud van, S84

Winter, Inge, S51, S74

Winterer, Georg, S343

Witte, Lot D., S82

Witte, Lot de, S117

Wittert, Gary, S284

Wo, Sui Fung, S214

Wobrock, Thomas, S343

Wojcik, Joanne, S235, S236

Wolf, Achim, S23

Wolf, Daniel, S57

Wölwer, Wolfgang, S343

Wong, Cheuk Fei, S214

Wong, Chloe, S25

Wong, Katie, S92

Wong, Keri, S333, S334

Wong, Kwok Tung Gordon, S208

Wong, Tien, S12

Woo, Tsung-Ung Wilson, S235, S236

Wood, Joel, S63

Wood, Stephen, S98, S144, S147, S209, S209, S280, S281, S316, S328, S334, S357, S370

Woodall, Tamara, S334

Woodberry, Kristen, S36, S54, S78, S366

Woods, Scott W., S78, S102, S231

Woods, Scott, S39, S62, S79, S266, S396, S422

Woods, Will, S138

Woodward, Melissa, S291

Woodward, Todd, S388

Woopen, Christiane, S285

Worbe, Yulia, S360

Wright, Abigail, S212

Wright, Stephen, S27

Wright-Martin, Jillian, S322

Wulff, Sanne, S424

Wulfman, René, S167

Wunderink, Alexander, S260

Wunderink, Lex, S238

Wykes, Til, S106, S196, S307

Xiang, Yu-Tao, S352

Xiao, Lan, S42, S297

Xin, Lijing, S15, S133, S236

Xiong, Lan, S352

Xu, Feng, S289

Xu, Junzhe, S188, S189

Xu, LiHua, S223, S366

Xu, Xin, S331

Xu, Zhida, S82

Yalcin, Nadir, S345

Yamagishi, Mika, S381

Yamaguchi, Taiju, S315

Yamamori, Hidenaga, S419

Yamasue, Hidenori, S277

Yan, Tingjian, S97, S248, S366

Yan, Wenjing, S170

Yanamori, Hidenaga, S85

Yang, Cheng, S331

Yang, Lawrence, S54

Yang, Rang, S129

Yang, So Yung, S362

Yang, Yen Kuang, S389

Yang, Ziyi, S63

Yasuda, Yuka, S419

Yasuyama, Toshiki, S248

Yazbek, Hanan, S359

Yazici, Mumin Kazim, S345

Yee, Jie Yin, S309

Yi, Jung-Seo, S403

Yi, Sang Hoon, S203

Yi, Zhen-hui, S358

Yildiz, Mustafa, S211, S283, S316, S419

Yoffe, Rinat, S23

Yohn, Samantha, S70, S127, S204

Yokokura, Masamichi, S277

Yolken, Robert H, S63

Yolken, Robert H., S150

Yolken, Robert, S62, S63, S68

Yolland, Caitlin, S252

Yoo, Taeyoung, S177

Yoon, Bo-Hyun, S161, S201, S206, S215, S218, S236, S264, S296, S304, S326, S401, S411

Yoon, Jin-Sang, S177, S411

Yoon, Woon, S219, S287, S387, S391

Yoon, Youngwoo, S186

York, Whitney, S425

Yoshida, Kazunari, S190

Yoshikawa, Etsuji, S277

Youn, Tak, S203

Young, Jared, S233

Yousefi, Vida, S344

Yu, Hua, S90, S394

Yu, Yaqin, S82

Yuan, Jianxin, S188

Yuan, Jiaxin, S189

Yuen, Hok Pan, S99, S116, S130, S132, S228, S326, S378

Yum, Jiyun, S307

Yung, Alison Ruth, S132

Yung, Alison, S99, S116, S156, S228, S312, S334, S378, S388

Zahid, Uzma, S118

Zahle, Kate Kold, S252

Zai, Clement, S115, S218, S399, S412

Zakskorn, Lauren, S3

Zakzanis, Konstantine, S306, S354

Zalesky, Andrew, S74, S91, S176, S282

Zammit, Stan, S325

Zammit, Stanley, S39, S48, S53, S72, S165, S247, S270, S273, S377, S379

Zanatta, Daniela, S166

Zandian, Arasch, S120

Zanelli, Jolanta, S77

Zang, Feifei, S331

Zaytseva, Yulia, S173

Zelaya, Fernando, S81, S382

Zenoozian, Saeedeh, S344

Zhand, Naista, S274

Zhang, Chengcheng, S121, S301

Zhang, Fan, S194

Zhang, Landan, S156

Zhang, Lei, S176

Zhang, Peter, S133

Zhang, TianHong, S36, S90, S104, S223, S366

Zhao, Liansheng, S90, S394

Zhao, Wei, S187

Zhening, Liu, S187

Zhou, Baiwan, S331

Zhou, Fuchun, S352

Zhou, Han-yu, S333

Zhou, Juan, S9, S281

Zhou, Ping, S352

Zhu, Xiyu, S75

Zia, Aqsa, S3

Zijl, Peter van, S289

Zink, Mathias, S371

Zipursky, Robert, S267, S415, S418

Zorzi, Carline, S389

Zouhaier, El Hechmi, S152

Zuardi, Antonio W., S297

Zuardi, Antonio Waldo, S28

Zubiaurre-Elorza, Leire, S347

Zugman, André, S184

Zugman, Andre, S286

Zunszain, Patricia, S118

Žarko,Bajić, S257, S257, S20, S136, S259, S275, S316

